# Supplementary material for: Defying decomposition: the curious case of choline chloride
Source: Nat Commun. 2023 Oct 21;14:6684. doi: 10.1038/s41467-023-42267-6 (PMC10590384; doi:10.1038/s41467-023-42267-6)
Supplement: Supplementary file 1 — SUPPLEMENTARY INFO [file 41467_2023_42267_MOESM1_ESM.pdf]

Supplementary Information for

Defying Decomposition: The Curious Case of Choline Chloride

van den Bruinhorst et al.

September 17, 2023

Contents

Supplementary Methods **2**
Materials and sample handling . . . 2
Fast differential scanning calorimetry . . . 3
Equipment . . . 3
Sample preparation . . . 3
Data acquisition and processing . . . 4
Sensor recovery . . . 6
Fast scanning calorimetry + μ-XRD . . . 7
Equipment . . . 7
Sample preparation . . . 9
Data acquisition and processing . . . 9
Sensor recovery . . . 10
Fast scanning calorimetry + optical microscopy . . . 11
Equipment . . . 11
Data acquisition and processing . . . 11
Fusion properties used in Fig. 1 . . . 11

Supplementary Discussion **12**
S–S transition of ChCl . . . 12
Sample mass determination . . . 13
Impact of fusion properties . . . 15

Supplementary Figures **19**

Supplementary Tables **29**

Supplementary Methods

Materials and sample handling

Choline chloride (ChCl) was purchased from Acros, the product purity is 99 % and the batch purity is 100.1 % from argentometric titration on a dry basis and it has a water content of 0.7 % (certificate of analysis). It was recrystallised from technical grade absolute ethanol, dried for at least 72 h under vacuum (< 0.3 mbar) and stirring, and finally stored under dry argon atmosphere.

ChCl was recrystallised under argon by saturating an absolute ethanol solution at 333 K ($w_{\mathrm{ChCl}} \approx 0.40$) in a 20 mL vial and letting it cool down slowly to approximately 301 K overnight, yielding 0.5 cm to 2 cm long clear crystalline needles/shards (Supplementary Figure 1). The saturated ethanol solution was decanted, the needles were washed with refrigerated absolute ethanol, and then dried in a clean vial at 333 K.

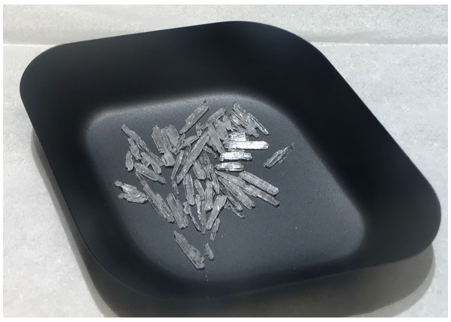

Supplementary Figure 1 Picture of the ChCl shards under argon atmosphere, after recrystallisation from ethanol.

All samples were handled under dry and inert atmosphere at all times (Supplementary Figure 2). At ENS de Lyon, the fast differential scanning calorimeter (FDSC) was placed in a Solocontainment soloLAB glovebag that was flushed with dry argon. Before being transported, ChCl was sealed in a vial under argon, which was then sealed in a larger vial under argon to create a double-layered protection against moisture uptake. At the ESRF, a stereo-microscope was placed in an Erlab XL pyramid glovebag to prepare the samples under inert N_2 atmosphere. Since this glovebag inhibits the connection of a power cable, a flashlight and a flat portable light source were used as reflection and transmission lights, respectively.

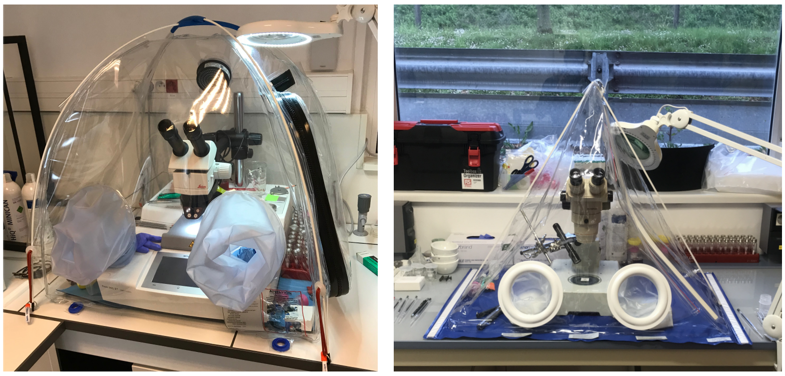

Supplementary Figure 2 Picture of **(left)** the fast differential scanning calorimeter in a glovebag at ENS Lyon, and **(right)** the stereo-microscope in a glovebag at ESRF.

Fast differential scanning calorimetry

Equipment

A Mettler-Toledo Flash-DSC 2+ equipped with a Leica Microsystems stereo-microscope (model Leica LED3000 RL, 58mm) with a total magnification of 40x was used for the Fast Differential Scanning Calorimetry (FDSC) measurements. The whole instrument was placed into a glovebag flushed with dry argon during sample preparation and FDSC operation. Two chip architectures were employed, the UFH 1 and the UFS 1 sensor [1]. Each sensor was conditioned and corrected according to the procedure defined by the manufacturer. The sample stage temperature was set to $303.15\,\mathrm{K}$.

Sample preparation

The deposited samples are in the order of $1\,\mathrm{ng}$ to $50\,\mathrm{ng}$, hence the samples were prepared under the FDSC stereo microscope. To handle such small samples, we used hairs from the brush supplied by the manufacturer or a thin metal wire taken from a capacitor. The hairs were installed on the pen supplied with the equipment, or taped to a regular pen extended with a pipette tip.

In order to improve the thermal and physical contact between the active sensor area and the ChCl particles, a thin film of silicon grease was employed prior to the placement of ChCl. Various silicon oils were explored to prevent the ChCl particles from *jumping* off the sensor area, a phenomenon that is common to FDSC [2]. Silicon oils with low viscosity did not retain the ChCl particles. Therefore, a highly viscous Korasilon silicon grease was deposited on the sensor by carefully touching a droplet of grease sitting on a glass slide with the tip of a hair, and subsequently gently spreading the grease on the centre of the sensor. The grease did not show any thermal events within the temperature range and at the heating rates under study. However, when the film was too thick, the heat flow signal was noisy at high rates ($>10\,000\,\mathrm{K{\cdot}s^{-1}}$) and the ChCl peaks could not be resolved. This was also the case when the particle was immersed in grease outside the sensor area and subsequently moved with a coating of grease towards the center of the sensor area. When in doubt about the suitability of the applied grease film thickness, we verified the response of the sensor + grease at $20\,000\,\mathrm{K{\cdot}s^{-1}}$ within the temperature range of the experiment. In case of noise, we cleaned the chip (section Sensor recovery) and deposited a thinner film of grease.

To obtain ChCl particles of the appropriate dimensions, a small crystal (approximately 1 mm^3 suffices) of ChCl was ground on a microscope glass slide using a small pestle. A particle of the appropriate size was selected by eye and picked up using a hair tip that was slightly wetted with silicon grease by touching a droplet of grease and removing the excess from the hair tip with the fingertips of a nitrile glove. The particle was then deposited to the centre of the sensor and centred if needed using a dry hair. The reference side of the chip was left empty, as it was practically impossible to deposit the same amount of grease as was present at the sample side.

When the ChCl particle was not fully decomposed during heating with FSC, its position was typically retained throughout the experiment (Supplementary Figure 26). The silicone-grease used when positioning the particle could retain it to a lesser extend when using the FDSC chips, the particle often moved within or outside the active sensor area. Particle movement (Fig. 2D and Video S1) affects the FDSC peak shape and is likely to be a main contributor to the uncertainty in $\Delta_{\mathrm{fus}}H_{\mathrm{m}}$. Another factor could be the lack of control regarding the ChCl particle shape. Hence, we are currently investigating options to produce ChCl crystallites with controlled shape and size.

Data acquisition and processing

Typically, ChCl was exposed to two different temperature programs: i) a heating–cooling cycle from ambient temperature to a temperature well below T_{d} at 1000 K·s^{-1} to obtain $\Delta_{\mathrm{trs}}H$ of the ChCl $\alpha \rightarrow \beta$ solid–solid transition (Supplementary Figure 3), and ii) a high-temperature cycle well above T_{d} at the heating/cooling rates listed in Supplementary Table 1 without applying a high-temperature isotherm (Supplementary Figure 4). The maximum temperature, heating rate, and cooling rate of the high-temperature cycle were varied for some samples (Supplementary Table 1 and Supplementary Figure 21). From $\Delta_{\mathrm{trs}}H$ and the molar enthalpy of the solid–solid transition ($\Delta_{\mathrm{trs}}H_{\mathrm{m}}$ =16.3 kJ·mol^{-1} [3]), the sample mass was estimated.

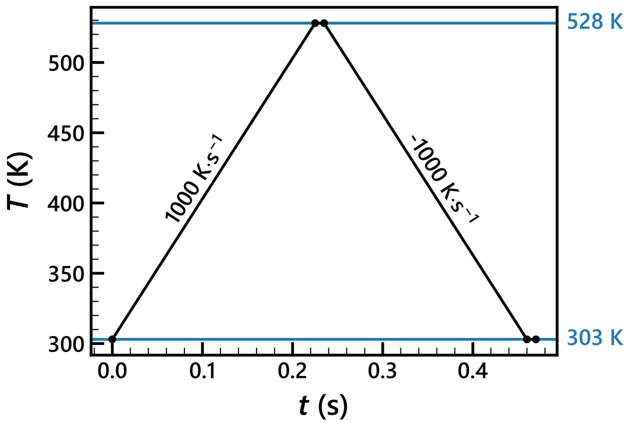

Supplementary Figure 3 Temperature program applied to determine $\Delta_{\mathrm{trs}}H$ of the ChCl $\alpha \rightarrow \beta$ solid–solid transition. The isotherms were 10 ms long.

For consecutive runs on the same ChCl particle, the solid often did not fully recrystallise to the α-solid and instead remained in the metastable β form under the applied conditions. This lead to an underestimation of the sample mass remaining on the sensor after heating above $T_{\alpha \rightarrow \beta}$. A reproducible sample mass could be obtained from the peak integral by ensuring full

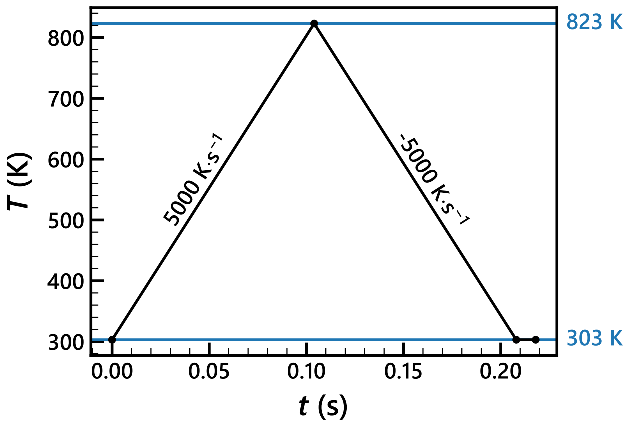

Supplementary Figure 4 Temperature program applied to determine $\Delta_{\mathrm{fus}}H_{\mathrm{m}}$ of ChCl. Note how no isotherm was applied at the maximum temperature.

recrystallisation to the stable α form. In between each consecutive run on the same ChCl particle, the following temperature program was repeated five times: ChCl was heated to 343.15 K at 1000 K·s^{-1} (approximately 10 K below T_{trs}), kept isothermally for 60 s, and cooled to 303.15 K at 1000 K·s^{-1} (Supplementary Figure 5).

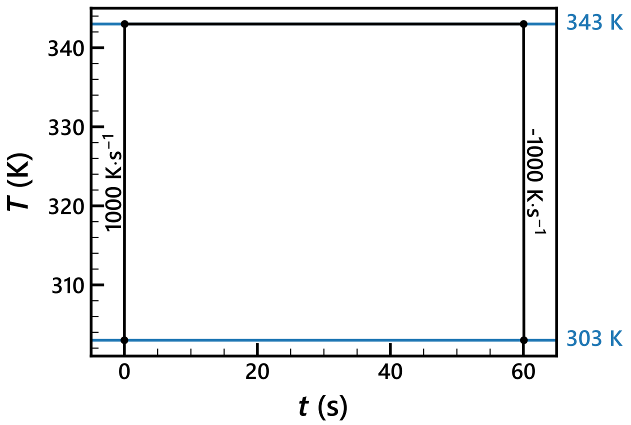

Supplementary Figure 5 Temperature program applied to recrystallise metastable β-ChCl to stable α-ChCl.

The heat flow signals were recorded using the Star-e software package, they were then integrated and visualised using python 3. $\Delta_{\mathrm{trs}}H$ was calculated from the integral of the solid–solid peak, which was numerically integrated using a sigmoidal baseline that scales with the peak integral. The start and end of the peak were determined by the point at which a rolling linear fit from the inflection point towards the baseline merges with the baseline. $\Delta_{\mathrm{fus}}H$ was determined in two ways: i) fitting all data to a melting + decomposition model, ii) fitting a single melting peak to the data until the inflection point after the first major peak.

Melting + decomposition Using this approach, we summed two Voigt profiles with a quadratic polynomial baseline fitted to a 100 K temperature range without thermal events (typically 550 K to 650 K). The first Voigt profile represents the melting of ChCl and the second profile represents all heat effects owing to decomposition (e.g. breaking of covalent bonds, evaporation of decomposition products). Since the decomposition involves mass-loss, the heat-flow is also affected by a loss of C_P. To account for this effect, the second peak is summed with its negatively scaled (numerical) cumulative distribution function; the amplitude was kept as a free fitting parameter, while σ, γ, and the peak centre were adopted from the decomposition peak. The amplitude of the mass loss could not be coupled to the amplitude of the decomposition peak directly, because the ratio of ChCl and Si-grease is different for each sample.

The Voigt model was implemented using the lmfit package [4]:

$$f(x, A, \mu, \sigma, \gamma) = \frac{A\Re[w(z)]}{\sigma\sqrt{2\pi}}, \tag{1}$$

where

$$z = \frac{x - \mu + i\gamma}{\sigma\sqrt{2\pi}} \tag{2}$$

and

$$w(z) = e^{-z^2}\mathrm{erfc}(-iz), \tag{3}$$

x is the independent data variable, A is the peak amplitude, μ is the peak centre, and σ and γ are constrained to each other and represent the standard deviation and scale parameter of the convoluted Gaussian and Lorentz distributions, respectively.

The peak integral or $\Delta_{\mathrm{fus}}H$ was obtained by integrating the first fitted Voigt profile from $-\infty$ to ∞. A visualisation of the fit and its components is given in Supplementary Figure 6, all fits of the melting + decomposition approach are shown in Supplementary Figure 20.

Single melting peak Using this approach, we fitted part of the data to a quadratic polynomial + single Voigt profile. The baseline was determined in the same temperature range as for the melting + decomposition approach. The data range to which the Voigt profile was fitted was determined by taking the numerical derivative of the heat flow signal and selecting the minimum (inflection point) after the first major peak. The peak integral or $\Delta_{\mathrm{fus}}H$ was obtained by integrating the fitted Voigt profile from $-\infty$ to ∞. A visualisation of the fit and its components is given in Supplementary Figure 6, all fits of the single melting peak approach are shown in Supplementary Figure 21.

Sensor recovery

The sensors were recovered by vertically submerging the chips in a 25 mL beaker with solvent. Three solvents were applied: distilled water to dissolve ChCl, ethanol to remove the water, and petroleum ether to remove the grease. The cleanliness of the sensors was verified visually under a stereo microscope as well as by the heat flow baseline from 303 K to 723 K at 1000 $\mathrm{K \cdot s^{-1}}$. ChCl was readily removed from the sensor area, but adequate removal of the grease typically required several subsequent washes with petroleum ether alternated with rubbing the sensor surface using a clean hair.

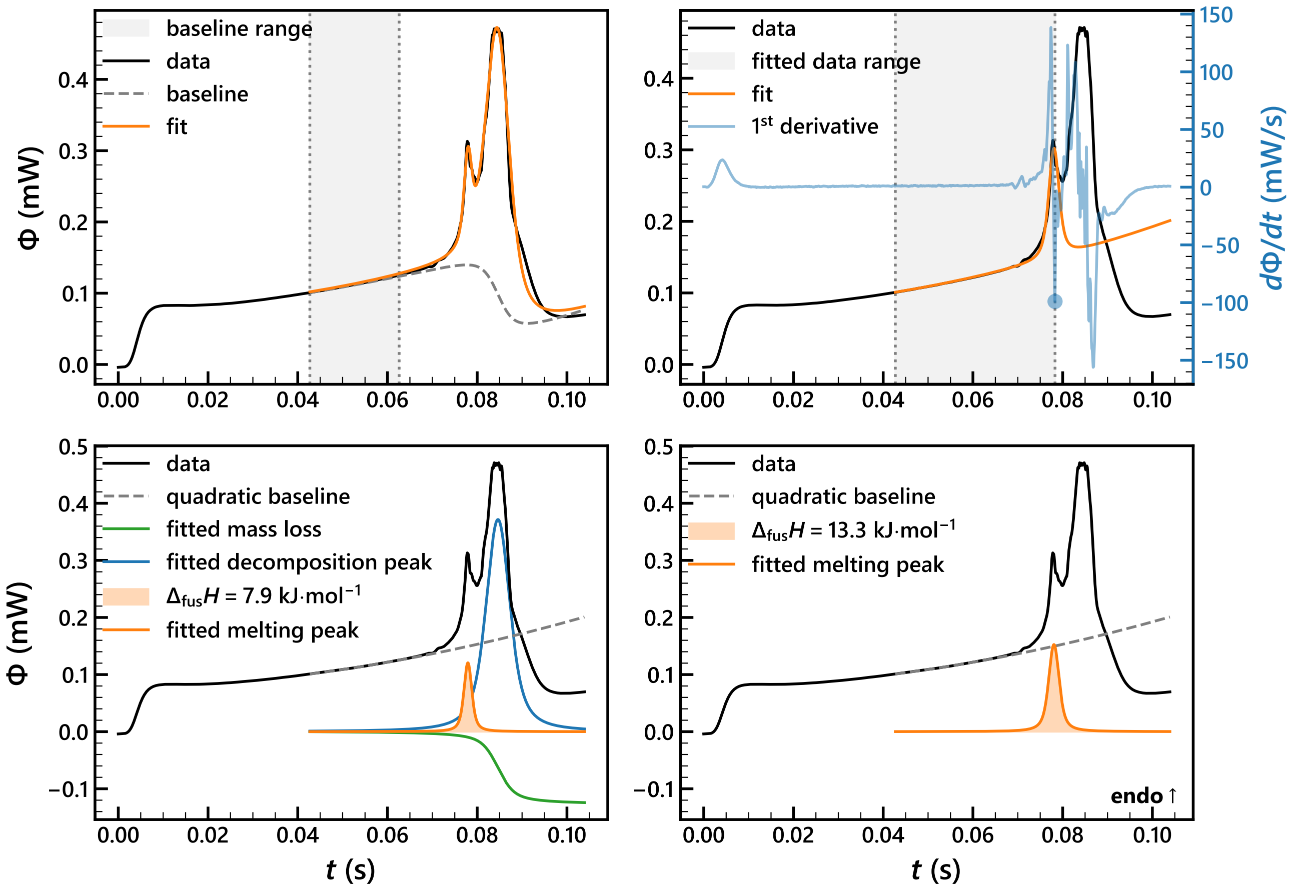

Supplementary Figure 6 High-temperature FDSC thermogram of peak no. 10 (black line, see also table 1). The data was fit using the melting + decomposition model (*left*) or a single Voigt profile (*right*) using the approach described in section Data acquisition and processing. *Top*: the model fit to the data (orange line) is shown together with the data range (grey area) used to fit quadratic baseline (left) or the Voigt profile (right). On the right, the first derivative of Φ is shown in blue and the minimum after the first major peak is highlighted (circle). *Bottom*: the components of each model are shown separately. The quadratic baseline (grey dashes) is the same for both models, while the melting peak and integral (orange line and area) are model specific. For the melting + decomposition model (*left*) the decomposition peak (blue line) and the heat flow response of the mass loss (green line) are shown as well.

Fast scanning calorimetry + μ-XRD

Equipment

Fast scanning calorimetry (FSC) measurements were combined with *in-situ* ms time-resolved μ-XRD at the ID13 beamline at the ESRF. A monochromatic X-ray beam with a photon energy of 13 keV and a photon flux of approximately 3x10^{12} photons per second was focused by means of compound refractive Beryllium lenses to a spot size of 2.5 μm in both directions at a location 40 mm upstream of the sample position. Possible beam induced sample damage was mitigated

by adjusting the effective beam size to the sample size. This reduces the flux density and distributes the energy deposited in the sample by the X-ray beam over the entire sample volume. The effective footprint of the beam at the sample position was adjusted to match the size of the smallest sample diameter used in the experiment, corresponding to approximately 15 µm.

The specimen—deposited in the central area of the FSC chip sensor—was positioned and aligned in the X-ray beam with help of a retractable on-axis microscope, prior to each measurement.

An EIGER 4M single photon counting area detector from Dectris AG (Switzerland) was employed for the data collection of the two-dimensional diffraction patterns. The detector was operated at 500 Hz, slightly below its maximum sampling frequency of 750 Hz to ensure a stable performance during the entire time of the experiment.

The FSC used for the *in-situ* experiments is a custom device implemented at the ID13 beamline in 2014[5,6] and was used to study fast structural transitions of polymers and energetic materials[7–9]. The sensor chips used in the *in-situ* experiments are XEN-39392 from Xensor Integration (NL). The sensor chip constitutes a SiN_x membrane of 1 µm thickness supported by a Si frame, the active area is 100 µm by 100 µm (Supplementary Figure 7). Six thermocouples connected in series are placed around the active area to provide a large scanning and observation field. Two pairs of resistive heaters, two main and two secondary heaters, are placed on the sides of the active area.

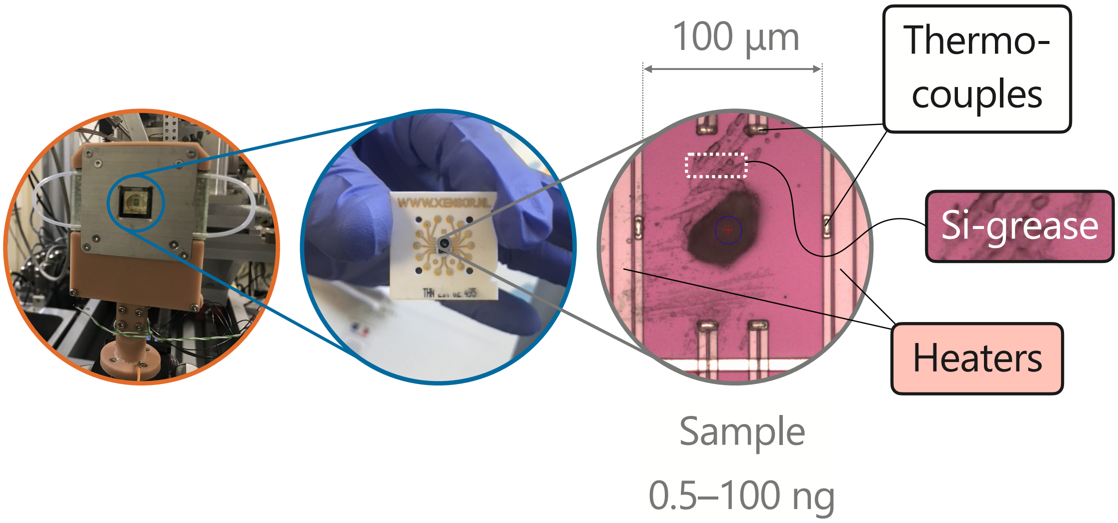

Supplementary Figure 7 From left to right: the hermetic chip holder in the XRD beam, a hand-held XEN-39392 chip, a close-up of the active sensor area with choline chloride particle and silicone grease.

While the main heater is used to apply the desired temperature program to the specimen, the secondary heater can be used to apply a temperature offset throughout the experiment. The offset shifts the temperature window to higher temperatures, in our case to 383 K to 730 K. When used in fast heating experiments, the FSC-device is operated in burst-mode where the desired temperature program is uploaded to the data acquisition board of the device prior to the experiment. After this "arming" procedure the temperature program is applied to the sensor upon a trigger signal while the thermal response signals from the sample are recorded.

The temperature in the centre of the active area is calibrated using the onset of the melting transition of four low melting metals, e.g. Indium, Tin, Bismuth and Zinc. The response time τ

of the sensor chip is estimated to be in the order of 4 ms derived from the exponential temperature response to a step-wise temperature change applied to the sensor when loaded with a typical sample.[8]

Sample preparation

ChCl was added to the sensor in the same fashion as described in section Sample preparation, except the sample size was typically larger than for the FDSC measurements. The sensor was then placed in a hermetic sample cell, using highly viscous Korasilon silicon grease as seal. The windows of the cell were SiN_x membranes with a thickness of 1 µm to ensure good transmission of the XRD signal. After sealing, the loaded sensor cell was removed from the glovebag and mounted on the cell holder in the working point of the µ-XRD setup as shown in Supplementary Figure 8.

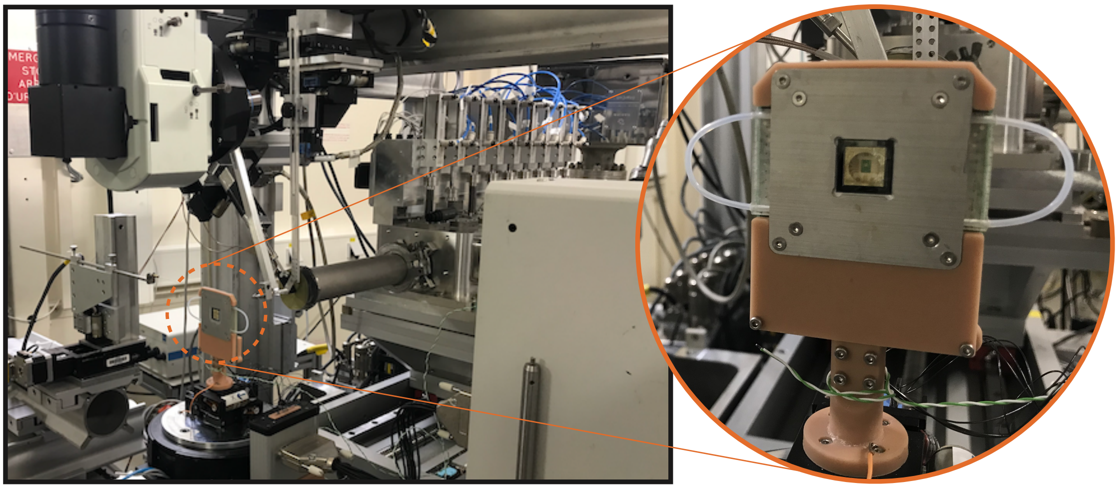

Supplementary Figure 8 Picture of the hermetic cell for the fast scanning calorimetry chip placed into the working point of the beamline setup, as well as a close-up of the same hermetic cell.

Data acquisition and processing

Prior to heating, the initial temperature of the FSC was set to approximately 383 K, i.e. above the $T_{\alpha\rightarrow\beta}$ transition by using the secondary heater on the FSC sensor. The secondary heater is switched off temporarily when arming the FSC-device, the sample temperature thus drops rapidly to ambient temperature prior to the fast heating run. Low-temperature α-ChCl is more sensitive to irradiation than high-temperature β-ChCl[10]. To avoid sample degradation, the time without temperature offset was kept as short as possible during the experiment, i.e. a few seconds.

A trigger scheme was implemented to ensure synchronisation of the µ-XRD data and the FSC data. The FSC triggered the activation of the fast X-ray shutter as well as the data collection of the Eiger4M detector. As a result, the sample would only be exposed to X-rays during the actual temperature program, reducing extensive exposure of the sample prior to and after FSC data collection.

A single temperature program was applied for each run (Supplementary Figure 9). The first isotherm of 25 ms allowed the furnace to stabilise at 383 K at the offset temperature before

heating at controlled rates. The sample was then heated to the maximum temperature ($\sim$730 K) at 100, 1000, 2000, or 5000 K·s^{-1} and kept isothermally for 10 ms before cooling as fast as possible (uncontrolled) to prevent decomposition.

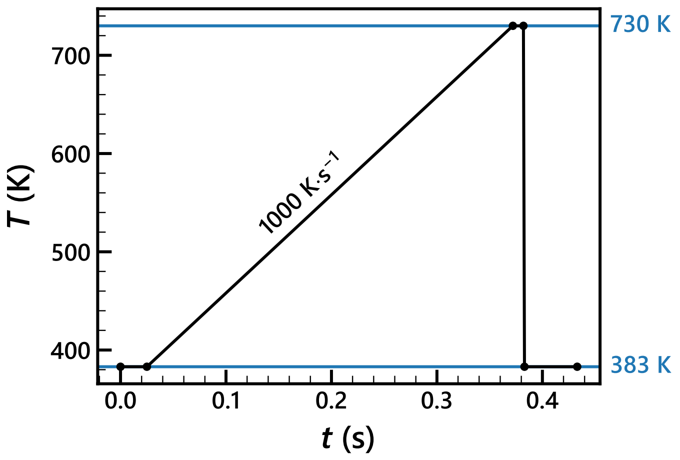

Supplementary Figure 9 Typical temperature program applied to the fast scanning calorimeter when doing *in-situ* µ-XRD measurements.

Before (and occasionally after) heating to high temperatures, a picture of the sample was taken using the retractable on-axis microscope. This allowed for the evaluation of any visual changes to the sample morphology owing to the temperature program and/or the X-ray beam exposure.

Typically, a single XRD-pattern (exposure time $\leq$ 20 ms) was collected before the heating run to verify the acquisition conditions as well as the sample position. While applying a temperature program (e.g. Supplementary Figure 9) XRD-patterns were recorded simultaneously every 2 ms.

The used diffraction geometry, including the modulus of the scattering vector q, was calibrated using several diffraction peaks of alpha-alumina. Here

$$q = \frac{4\pi}{\lambda} \sin\left(\frac{\theta}{2}\right),$$

where θ corresponds to the diffraction angle and λ is the wavelength of the used monochromatic X-ray beam. The collected two-dimensional diffraction patterns were resampled and reduced to one-dimensional scattering curves using the pyFAI package.[11–13] Further data reduction, processing and visualisation was done using custom protocols developed in Python 3.

Sensor recovery

While ChCl could be readily removed by submerging the sensor in water, exposure to the X-ray beam complicated the removal of the grease. The chips could not be fully recovered despite various efforts to clean the grease, e.g. flushing the sensor area with a stream of petroleum ether instead of submerging it. Hence, a pristine sensor was used for each measurement.

Fast scanning calorimetry + optical microscopy

Equipment

FSC measurements were combined with *in-situ* high-speed optical microscopy imaging. To this end, an Olympus BX50 optical microscope was equipped with a Phantom v7.3 fast CCD-camera from Vision Research US with a maximum frame rate of 15 kHz. The measurements were done using bright field transmission and reflection light illumination taking advantage of the good transparency of the SiN_x membranes.

The FSC sensor chips, sample cell, calorimeter, and glovebag were the same as those used for the μ-XRD experiments. Instead of placing the sealed sample cell in the μ-XRD beam, we placed it under a microscope equipped with high-speed camera.

Data acquisition and processing

To synchronise the fast heating measurements with the image acquisition, the fast CCD was triggered directly by the FSC device at the beginning of each heating run. Individual images were recorded at a fixed frame rate of 5 kHz.

Fusion properties used in Fig. 1

In order to construct Fig. 1 in the main manuscript, the following fusion properties were adopted for model component A: $T_{\mathrm{fus,A}} = 420\,\mathrm{K}$, $\Delta_{\mathrm{fus}}H_{\mathrm{m,A}} = 8\,\mathrm{kJ\cdot mol^{-1}}$. For ChCl the following properties were adopted from literature: $T_{\mathrm{fus,ChCl}} = 597\,\mathrm{K}$[14], $T_{\mathrm{trs}} = 352.2\,\mathrm{K}$[3], $\Delta_{\mathrm{trs}}H_{\mathrm{m}} = 16.3\,\mathrm{kJ\cdot mol^{-1}}$[3]. Finally, $T_{\mathrm{operate}} = 323\,\mathrm{K}$. For all indirectly derived fusion properties in literature, see Supplementary Table 5

Supplementary Discussion

S–S transition of ChCl

At ambient temperatures well below T_{trs}, β-ChCl would not readily recrystallise into the α-phase, showing no transition at all for particles weighing less than 50 ng. This is relevant as we use the integral of the S–S transition as a measure of the sample mass, and thus $\Delta_{fus}H_m$ or the degree of decomposition. Touching the metastable β-ChCl particles with the tip of a hair consistently induced the phase transition, but could also alter the sample mass—rendering the mass determination from the S–S transition unreliable.

Supplementary Figure 10 shows both: i) without touching the particle, ChCl would remain metastable in the β-phase such that the S–S transition would not occur and a flat line is measured. After touching, the peak consistently reappeared. ii) During the initial touch, the mass of the particle was slightly lowered, the consequent measurements were consistent, but mass loss due to touching the particle cannot be excluded.

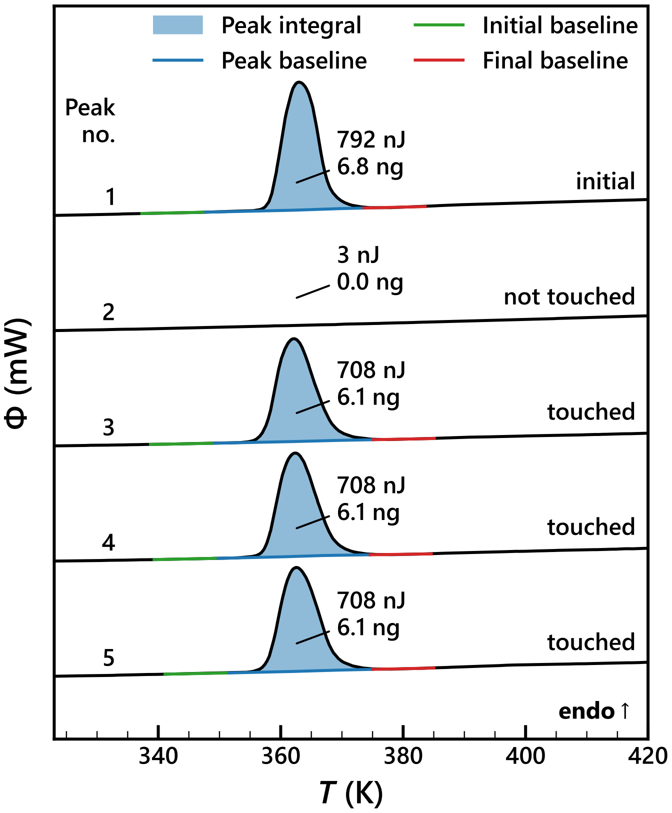

Supplementary Figure 10 Fast differential scanning calorimetry traces of successive heating–cooling cycles of choline chloride (only heating is shown) between 303 K and 523 K at 1000 K·s^{-1}. The integrals and derived sample mass are shown, as well as whether the particle was touched with a hair before heating.

Instead, a relatively long (300 s, or 5 times 60 s because of software limitations) isotherm was applied at 343 K, close to T_{trs}. This yielded reproducible S–S transition temperatures and enthalpies.

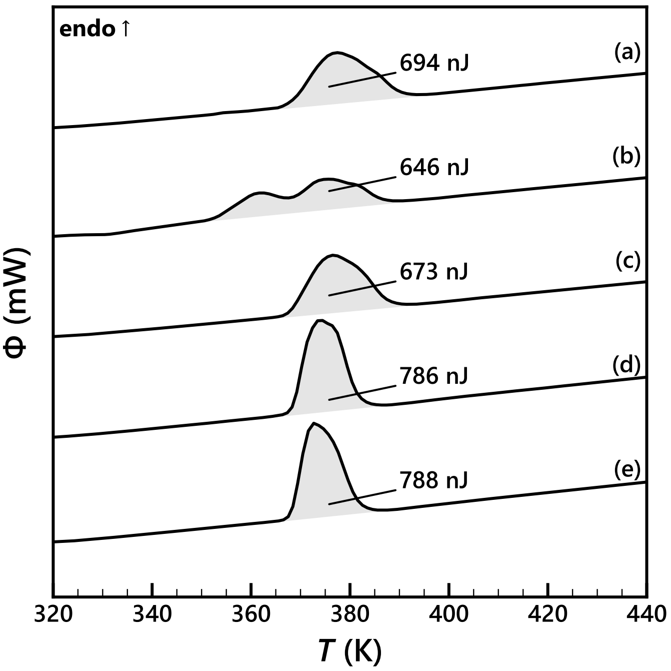

Supplementary Figure 11 Fast differential scanning calorimetry traces of successive heating–cooling cycles of a choline chloride particle (only heating is shown) between 303 K and 523 K at 1000 K·s^{-1}: (a) after sample deposition, (b) after one cycle and 5 isotherms of 1 min at 343 K, (c) after two cycles and a 30 min isotherm at 303 K, (d) after three cycles and a 12 h isotherm at 303 K, and (e) after four cycles and 5 isotherms of 1 min at 343 K.

Sample mass determination

Following the approach of Abdelaziz *et al.*[15], the mass can be determined from the ChCl heat capacity ($C_P(T)$) measured by FDSC and the molar heat capacity ($C_{P,\mathrm{m}}(T)$) reported in literature[3] from high-precision techniques:

$$\Phi_\mathrm{h}(T) = C_P(T) * \frac{dT}{dt}_\mathrm{h} + \Phi_\mathrm{loss}(T) \tag{4a}$$

$$\Phi_\mathrm{c}(T) = C_P(T) * \frac{dT}{dt}_\mathrm{c} + \Phi_\mathrm{loss}(T), \tag{4b}$$

where Φ is the heat flow (J·s^{-1}), $C_P(T)$ is the heat capacity (J·K^{-1}), and dT/dt is the heating rate (K·s^{-1}). Subscripts h, c, and loss denote heating, cooling, and the calorimeter and sample specific heat loss, respectively. When taking the heat flow of a section where the heating/cooling rate is controlled and $dT/dt_\mathrm{h} = -dT/dt_\mathrm{c}$, the heat loss is the same at each temperature and $C_P(T)$ can be determined by subtracting the two equations above:

$$C_P(T) = \frac{\Phi_\mathrm{h}(T) - \Phi_\mathrm{c}(T)}{2\frac{dT}{dt}_\mathrm{h}} \tag{5}$$

The mass can then be calculated from:

$$m = \frac{C_P(T) M_\mathrm{w}}{C_{P,\mathrm{m}}(T)}, \tag{6}$$

where m is the sample mass and M_w is the molar mass. Since we used silicon grease to improve the physical and thermal contact of the ChCl particle with the sensor, we corrected for the grease's heat capacity based on a grease-only run using the exact same temperature program prior to placing the ChCl particle:

$$C_{P,\mathrm{ChCl}}(T) = C_{P,\mathrm{grease+ChCl}}(T) - C_{P,\mathrm{grease}}(T). \tag{7}$$

We used a heating rate of $1000\,\mathrm{K{\cdot}s^{-1}}$ and we selected a temperature range where the heating rate is constant, the temperature difference between the grease and grease+ChCl is negligible, and the ChCl would be in the β-phase for which (extrapolated) $C_{P,\mathrm{ChCl,m}}$ data is available.

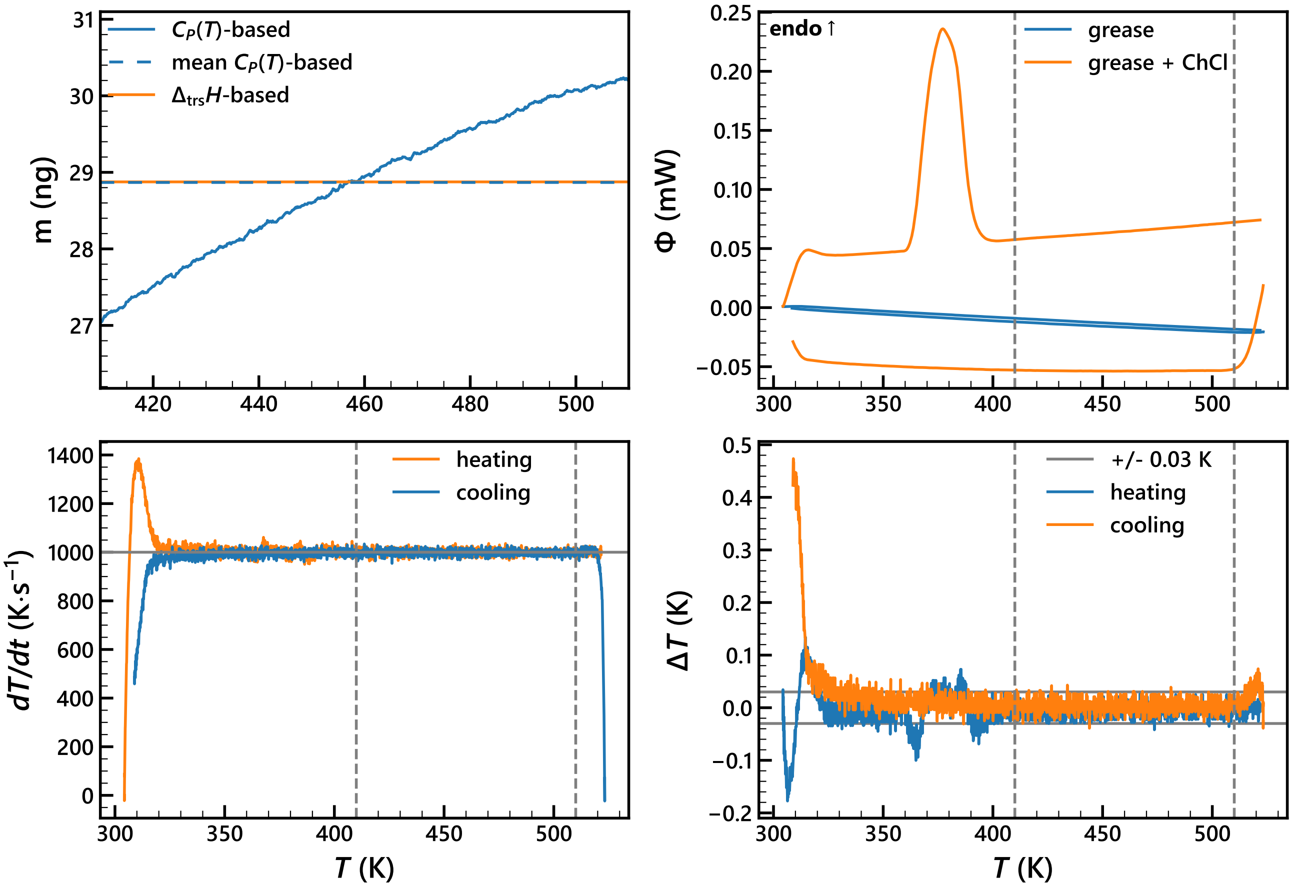

Supplementary Figure 12 Sample mass m (*top left*), heat flow Φ (*top right*), heating rate dT/dt (*bottom left*), and temperature difference between grease only and grease + ChCl using the same temperature program ΔT (*bottom right*) as a function of the sample temperature T. The temperature window that was used for the calculation of m from $C_P(T)$ is highlighted (grey dashes).

Supplementary Figure 12 shows that the particle mass calculated from $C_P(T)$ increases with temperature, which was reported in literature[15], albeit to a lesser extent. The mean of the $C_P(T)$-based m equals that calculated from $\Delta_{\mathrm{trs}}H$ and $\Delta_{\mathrm{trs}}H_{\mathrm{m}}$, confirming the validity of either method. $\Delta_{\mathrm{trs}}H$ can be obtained on heating over a smaller temperature range; neither a cooling curve nor a separate grease run are required. Hence, we preferred to determine m and $\Delta_{\mathrm{fus}}H_{\mathrm{m}}$ from $\Delta_{\mathrm{trs}}H$ rather than from $C_P(T)$.

Impact of fusion properties

Fig. 4 in the main text shows the impact of the new values for $\Delta_{\mathrm{fus}}H_{\mathrm{m}}$ on the thermodynamic analysis of the urea + choline chloride phase diagram. In this section we show the same analysis for $\Delta_{\mathrm{fus}}H_{\mathrm{m}} = 13.2 \pm 4.1\,\mathrm{kJ{\cdot}mol^{-1}}$, with and without taking $\Delta C_{P,\mathrm{m}}$ for the $\alpha \to \beta$ and melting transition into account. In order to account for $\Delta_{\mathrm{trs}}C_{P,\mathrm{m}}$ and $\Delta_{\mathrm{fus}}C_{P,\mathrm{m}}$ two thermodynamic cycles (Supplementary Figure 13) need to be considered: one for $T \leq T_{\mathrm{trs}}$ and one for $T_{\mathrm{trs}} < T$.

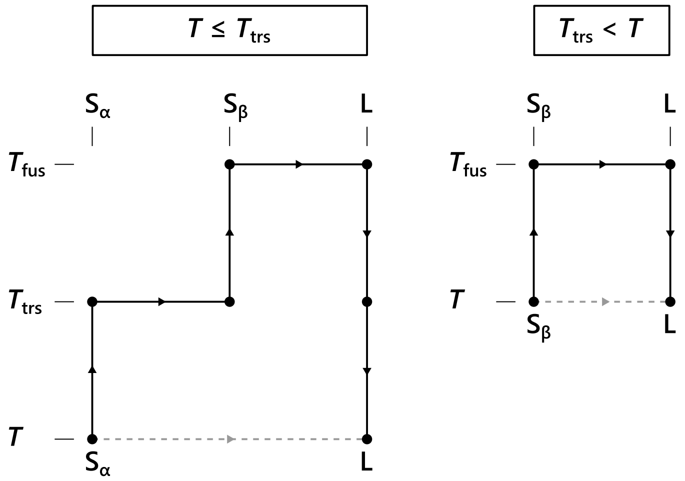

Supplementary Figure 13 Thermodynamic cycles for the solid (S) to liquid (L) transition at **(left)** liquidus temperatures (T) at and below the $\alpha \to \beta$ transition temperature (T_{trs}) or **(right)** T above T_{trs} of a simple choline chloride-based eutectic mixture, i.e. complete miscibility in L and complete immiscibility in S.

When assuming a simple eutectic mixture—which is complete miscibility in the liquid and complete immiscibility in the solid—the activity or partial molar excess Gibbs energy of ChCl ($G^{\mathrm{E}}_{\mathrm{m,ChCl}}$) can be related to the molar Gibbs energy of the liquidus phase transition ($\Delta^{\mathrm{L}}_{\mathrm{S}}G_{\mathrm{m}}$):

$$RT \ln x\gamma = RT \ln x + G^{\mathrm{E}}_{\mathrm{m,ChCl}} = -\Delta^{\mathrm{L}}_{\mathrm{S}}G_{\mathrm{m}}, \tag{8}$$

where x is the ChCl mole fraction and γ the activity coefficient of ChCl. The Gibbs energy of the liquidus phase transition at $T_{\mathrm{trs}} < T$ is:

$$\Delta^{\mathrm{L}}_{\mathrm{S_\beta}}G_{\mathrm{m}} = \Delta^{\mathrm{L}}_{\mathrm{S_\beta}}H_{\mathrm{m}} - T\Delta^{\mathrm{L}}_{\mathrm{S_\beta}}S_{\mathrm{m}}. \tag{9}$$

Following the thermodynamic cycle in Supplementary Figure 13, this can be rewritten in terms of $\Delta_{\mathrm{fus}}H_{\mathrm{m}}$ and the heat capacity of $\mathrm{S_\beta}$ and L:

$$\Delta^{\mathrm{L}}_{\mathrm{S_\beta}}H_{\mathrm{m}} = \int_{T}^{T_{\mathrm{fus}}} C^{\mathrm{S_\beta}}_{P,\mathrm{m}}(T)dT + \Delta_{\mathrm{fus}}H_{\mathrm{m}} + \int_{T_{\mathrm{fus}}}^{T} C^{\mathrm{L}}_{P,\mathrm{m}}(T)dT. \tag{10}$$

It is more convenient to write this in terms of $\Delta^{\mathrm{L}}_{\mathrm{S}_\beta} C_{P,\mathrm{m}}(T) = C^{\mathrm{L}}_{P,\mathrm{m}}(T) - C^{\mathrm{S}_\beta}_{P,\mathrm{m}}(T)$. Also, $C_{P,\mathrm{m}}$ data is often not available over the whole temperature range, especially since the L is metastable below T_{fus}, even if ChCl would not decompose at those temperatures. When taking $\Delta^{\mathrm{L}}_{\mathrm{S}_\beta} C_{P,\mathrm{m}}$ into account, it is therefore often assumed that the difference is constant with temperature. The integrals in (10) then simplify to:

$$\Delta^{\mathrm{L}}_{\mathrm{S}_\beta} H_{\mathrm{m}} = \Delta_{\mathrm{fus}} H_{\mathrm{m}} + \Delta_{\mathrm{fus}} C_{P,\mathrm{m}} \left(T - T_{\mathrm{fus}}\right). \tag{11}$$

Similarly,

$$\Delta^{\mathrm{L}}_{\mathrm{S}_\beta} S_{\mathrm{m}} = \frac{\Delta_{\mathrm{fus}} H_{\mathrm{m}}}{T_{\mathrm{fus}}} + \Delta_{\mathrm{fus}} C_{P,\mathrm{m}} \ln \frac{T}{T_{\mathrm{fus}}}, \tag{12}$$

which then gives:

$$\Delta^{\mathrm{L}}_{\mathrm{S}_\beta} G_{\mathrm{m},i} = \Delta_{\mathrm{fus}} H_{\mathrm{m}} \left(1 - \frac{T}{T_{\mathrm{fus}}}\right) + \Delta_{\mathrm{fus}} C_{P,\mathrm{m}} \left(T - T_{\mathrm{fus}} + T \ln \frac{T_{\mathrm{fus}}}{T}\right). \tag{13}$$

For $T \leq T_{\mathrm{trs}}$ the same approach can be taken, but the thermodynamic cycle should include the $\alpha \to \beta$ transition. Taking the integration limits of $\Delta C_{P,\mathrm{m}}(T)$ into account for both parts of the cycle:

$$\begin{aligned}\Delta^{\mathrm{L}}_{\mathrm{S}_\alpha} H_{\mathrm{m}} =& \Delta_{\mathrm{trs}} H_{\mathrm{m}} + \Delta^{\mathrm{L}}_{\mathrm{S}_\alpha} C_{P,\mathrm{m}} \left(T - T_{\mathrm{trs}}\right) + \\ & \Delta_{\mathrm{fus}} H_{\mathrm{m}} + \Delta_{\mathrm{fus}} C_{P,\mathrm{m}} \left(T_{\mathrm{trs}} - T_{\mathrm{fus}}\right).\end{aligned} \tag{14}$$

We assumed that $\Delta C_{P,\mathrm{m}}$ is independent of T, thus:

$$\Delta^{\mathrm{L}}_{\mathrm{S}_\alpha} C_{P,\mathrm{m}} = \Delta_{\mathrm{trs}} C_{P,\mathrm{m}} + \Delta_{\mathrm{fus}} C_{P,\mathrm{m}}. \tag{15}$$

Combining (15) with (14) and taking the same approach for $\Delta^{\mathrm{L}}_{\mathrm{S}_\alpha} S_{\mathrm{m}}$, we find:

$$\begin{aligned}\Delta^{\mathrm{L}}_{\mathrm{S}_\alpha} G_{\mathrm{m},i} =& \Delta_{\mathrm{trs}} H_{\mathrm{m}} \left(1 - \frac{T}{T_{\mathrm{trs}}}\right) + \Delta_{\mathrm{trs}} C_{P,\mathrm{m}} \left(T - T_{\mathrm{trs}} + T \ln \frac{T_{\mathrm{trs}}}{T}\right) + \\ & \Delta_{\mathrm{fus}} H_{\mathrm{m}} \left(1 - \frac{T}{T_{\mathrm{fus}}}\right) + \Delta_{\mathrm{fus}} C_{P,\mathrm{m}} \left(T - T_{\mathrm{fus}} + T \ln \frac{T_{\mathrm{fus}}}{T}\right).\end{aligned} \tag{16}$$

Recently, Lobo Ferreira *et al.*[3] measured $\Delta_{\mathrm{trs}} C_{P,\mathrm{m}} = 20 \pm 3\,\mathrm{J \cdot mol^{-1} \cdot K^{-1}}$ and estimated $\Delta^{\mathrm{L}}_{\mathrm{S}_\alpha} C_{P,\mathrm{m}} = 39.3 \pm 10.0\,\mathrm{J \cdot mol^{-1} \cdot K^{-1}}$ by extrapolating the $C_{P,\mathrm{m}}$ of aqueous solutions of ChCl to pure ChCl. From (15), we calculated $\Delta_{\mathrm{trs}} C_{P,\mathrm{m}} = 19.3 \pm 10.4\,\mathrm{J \cdot mol^{-1} \cdot K^{-1}}$, where the uncertainties are all within a 95% probability interval.

Supplementary Figures 14 and 15 show the thermodynamic analysis of the eutectic phase diagram of urea + ChCl for $\Delta_{\mathrm{fus}} H_{\mathrm{m}} = 13.2 \pm 4.1\,\mathrm{kJ \cdot mol^{-1}}$ or $\Delta_{\mathrm{fus}} H_{\mathrm{m}} = 13.8 \pm 3.0\,\mathrm{kJ \cdot mol^{-1}}$ compared to $\Delta_{\mathrm{fus}} H_{\mathrm{m}} = 4.3 \pm 0.6\,\mathrm{kJ \cdot mol^{-1}}$, with and without taking $\Delta C_{P,\mathrm{m}}$ for the $\alpha \to \beta$ and melting transition into account.

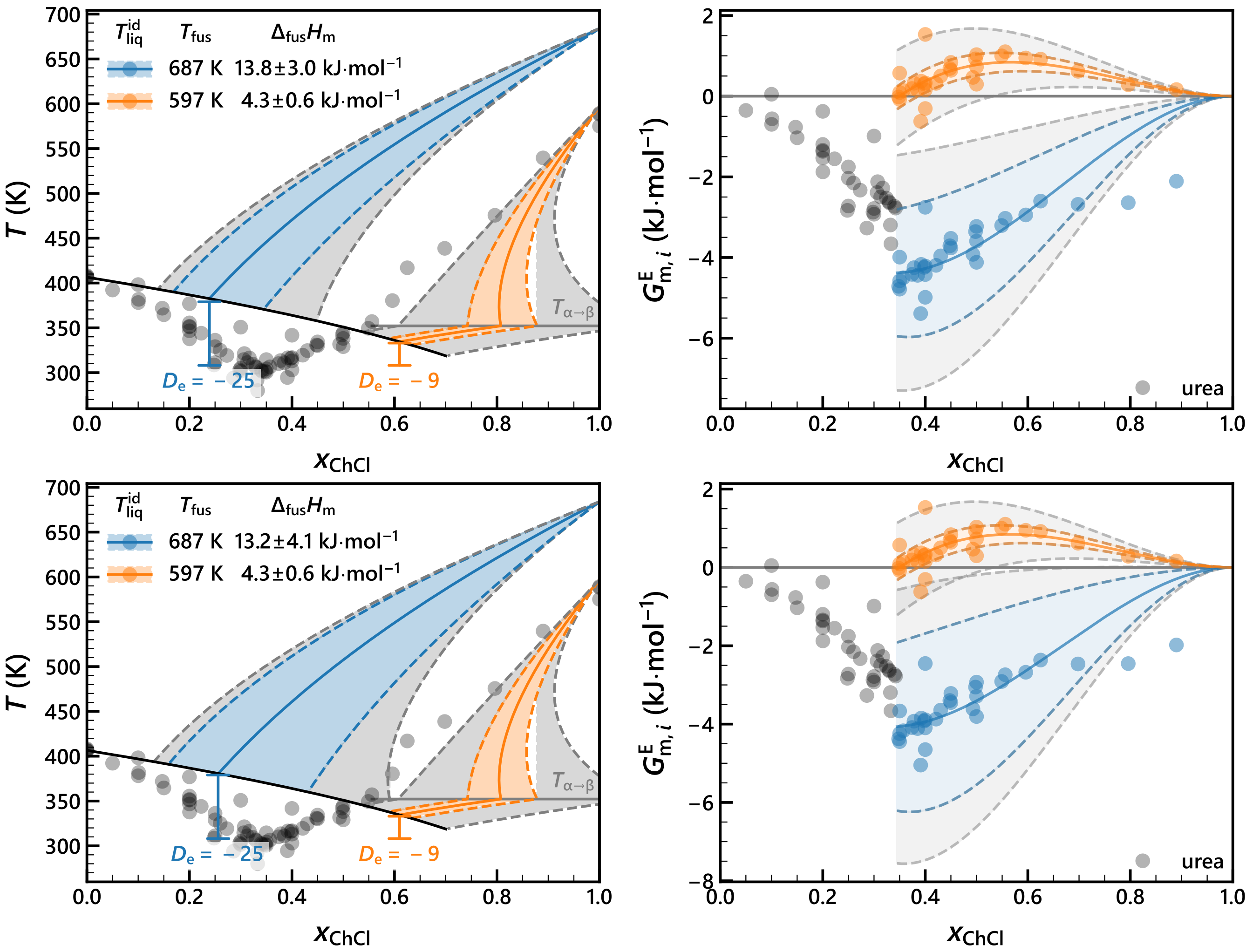

Supplementary Figure 14 (**left**) Experimental liquidus temperatures for urea + choline chloride from literature[16] (black circles) with the ideal liquidus temperature ($T_{\rm liq}^{\rm id}$, solid lines) calculated using different values for the ChCl enthalpy of fusion ($\Delta_{\rm fus}H_{\rm m}$) and their 95% confidence interval (coloured area between coloured dashed lines), taking into account $\Delta_{\rm fus}C_{P{\rm m,ChCl}} = 19.3 \pm 10.4\,{\rm kJ{\cdot}mol^{-1}}$ and $\Delta_{\rm trs}C_{P{\rm m,ChCl}} = 20 \pm 3\,{\rm kJ{\cdot}mol^{-1}}$[3] and their added 95% confidence interval (grey area between grey dashed lines). The eutectic depth ($D_{\rm e}$) is shown for both $\Delta_{\rm fus}H_{\rm m}$ values. (**right**) Partial molar excess Gibbs energy ($G_{{\rm m},i}^{\rm E}$) derived from the experimental liquidus temperatures and $\Delta_{\rm fus}H_{\rm m}$, $T_{\rm fus} = 597\,{\rm K}$[14]. Lines are fitted to $G_{{\rm m},i}^{\rm E}$ using a 2-parameter Redlich–Kister polynomial, the dashed lines and shaded areas correspond to the 95% confidence interval of $\Delta_{\rm fus}H_{\rm m}$ (coloured), and $\Delta C_{P{\rm m,ChCl}}$ (grey).

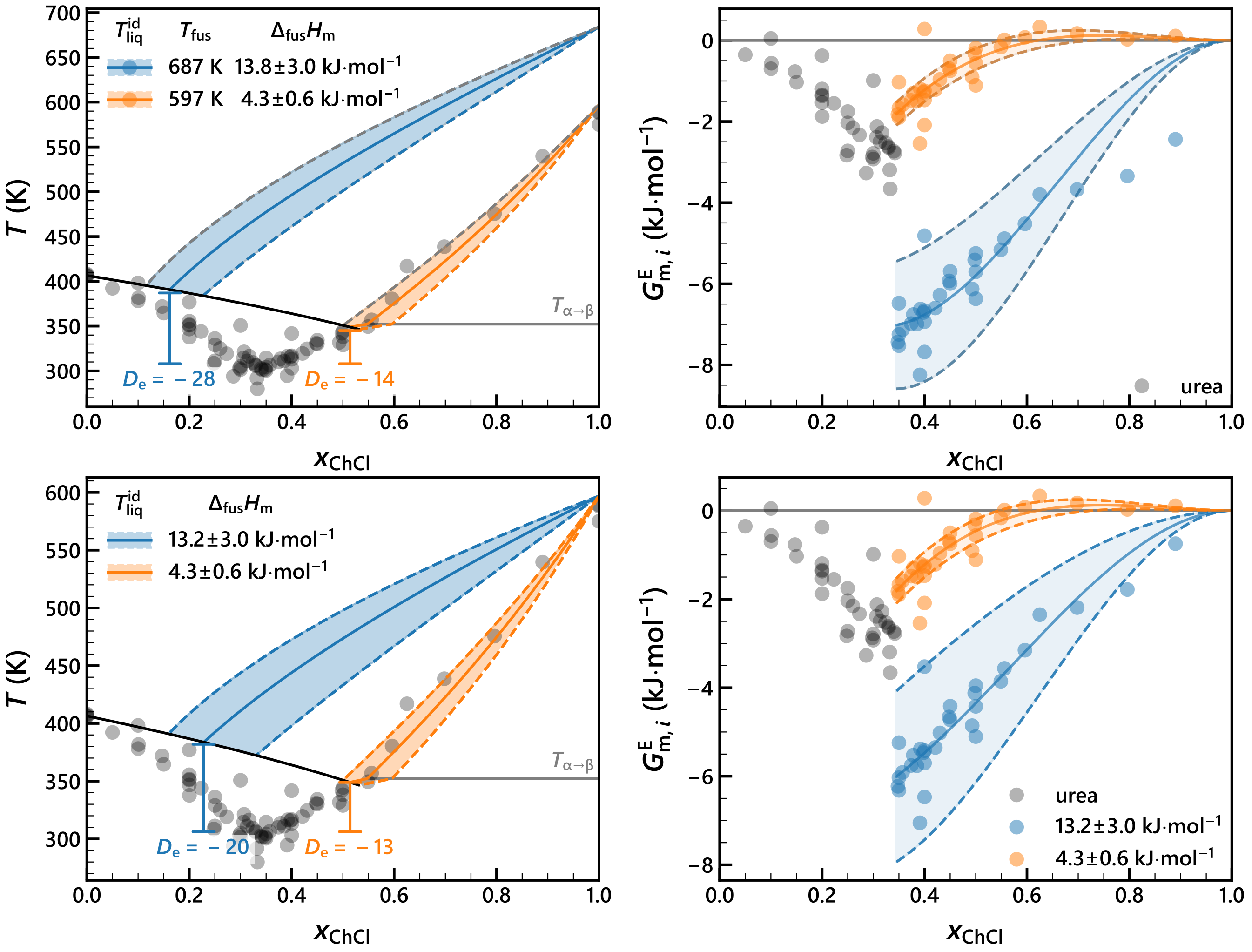

Supplementary Figure 15 (**left**) Experimental liquidus temperatures for urea + choline chloride from literature[16] (black circles) with the ideal liquidus temperature (T_{liq}^{id}, solid lines) calculated using different values for the ChCl enthalpy of fusion ($\Delta_{fus}H_m$) and their 95% confidence interval (coloured area between coloured dashed lines), not taking into account $\Delta_{fus}C_{P\mathrm{m,ChCl}}$ or $\Delta_{trs}C_{P\mathrm{m,ChCl}}$. The eutectic depth ($D_e$) is shown for both $\Delta_{fus}H_m$ values. (**right**) Partial molar excess Gibbs energy ($G_{m,i}^E$) derived from the experimental liquidus temperatures and $\Delta_{fus}H_m$, T_{fus} = 597 K[14]. Lines are fitted to $G_{m,i}^E$ using a 2-parameter Redlich–Kister polynomial, the dashed lines and shaded areas correspond to the 95% confidence interval of $\Delta_{fus}H_m$.

Supplementary Figures

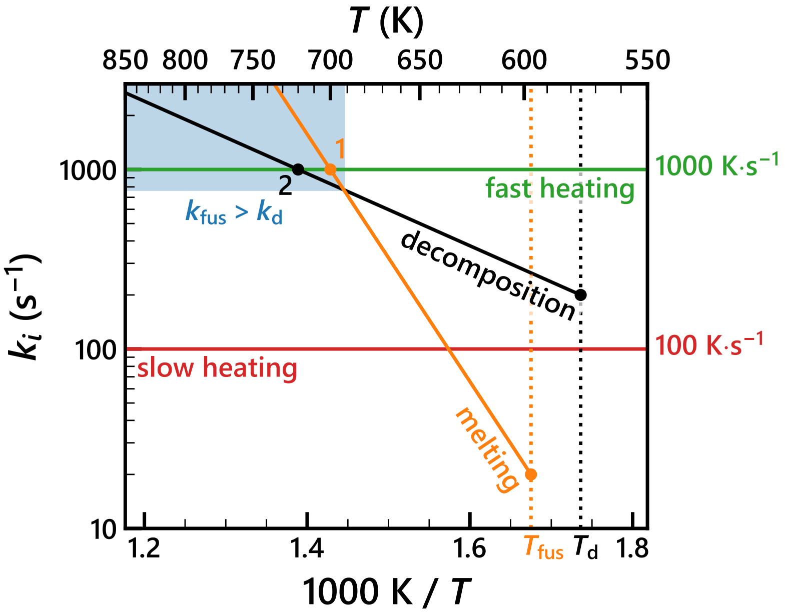

Supplementary Figure 16 Hypothetical activation diagram for choline chloride, adapted from Abdelaziz *et al.*[15]. Shown are the rate of melting (solid orange line), rate of decomposition (solid black line), heating rates of 100 K·s^{-1} (red line) and 1000 K·s^{-1} (green line), the melting point (T_{fus}, dotted orange line), decomposition temperature (T_d, dotted black line), and the area where the rate of melting is higher than the rate of decomposition ($k_{fus} > k_d$, blue shade). The order of transitions are highlighted for a heating rate of 1000 K·s^{-1}: first melting (1) and then decomposition (2).

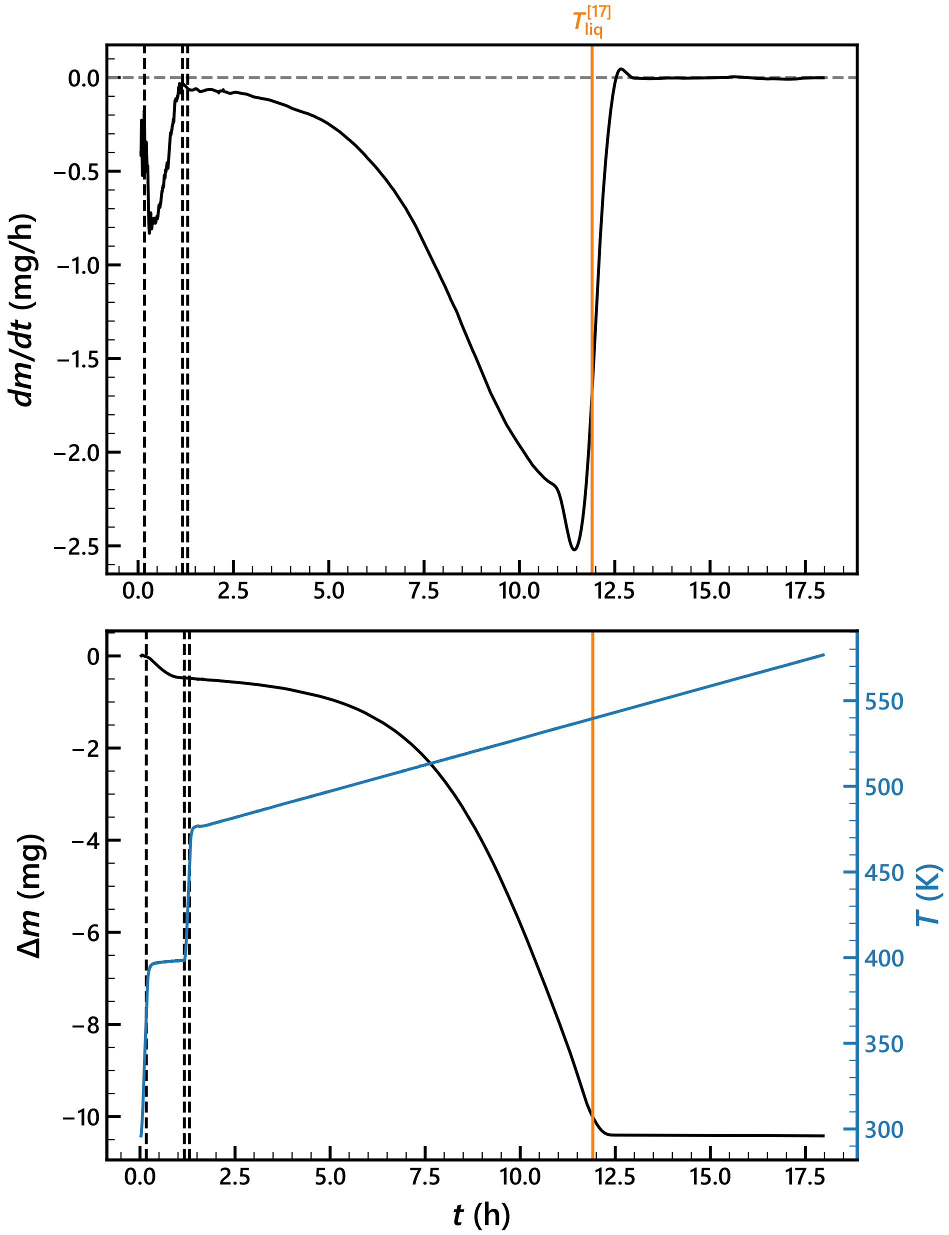

Supplementary Figure 17 Thermogravimetric analysis of urea + ChCl $x_{\mathrm{ChCl}} = 0.9$ using the same conditions as in Silva et al. [17], heating from 473 K to 573 K at a rate of 0.1 K·min^{-1} (1.67 mK·s^{-1}) after a drying step at 393 K for 1 h. Changes in heating rate are marked with a vertical dashed line. Shown are **(top)** the smoothed mass loss rate (dm/dt) over time (t) and **(bottom)** the mass loss (Δm, black line) and sample temperature (T, blue line) over time. The reported liquidus temperature $T_{\mathrm{liq}} = 539.6$ K [17] is highlighted in orange.

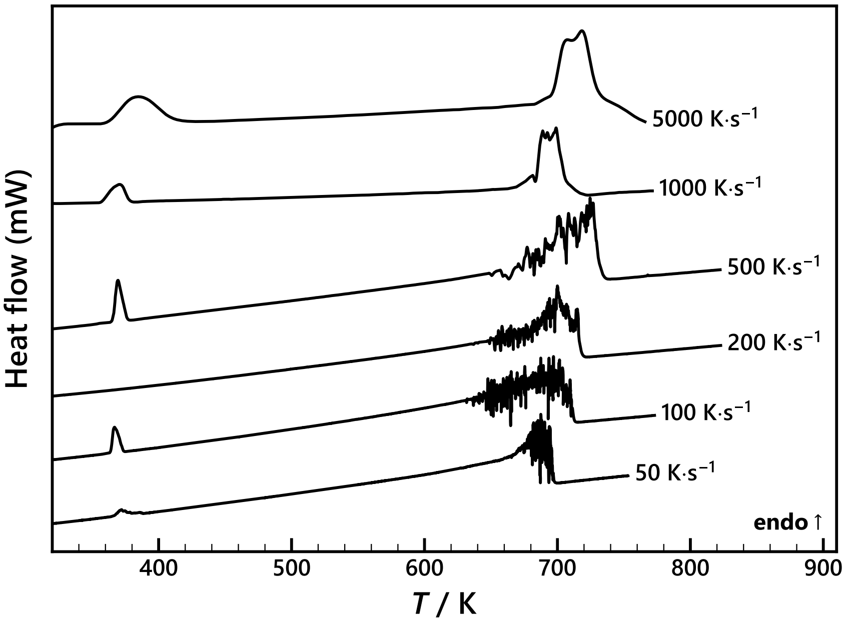

Supplementary Figure 18 FDSC thermograms (offset for clarity) of ChCl particles heated at several rates. For heating rates $<1\,\mathrm{kK{\cdot}s^{-1}}$ complete decomposition was observed.

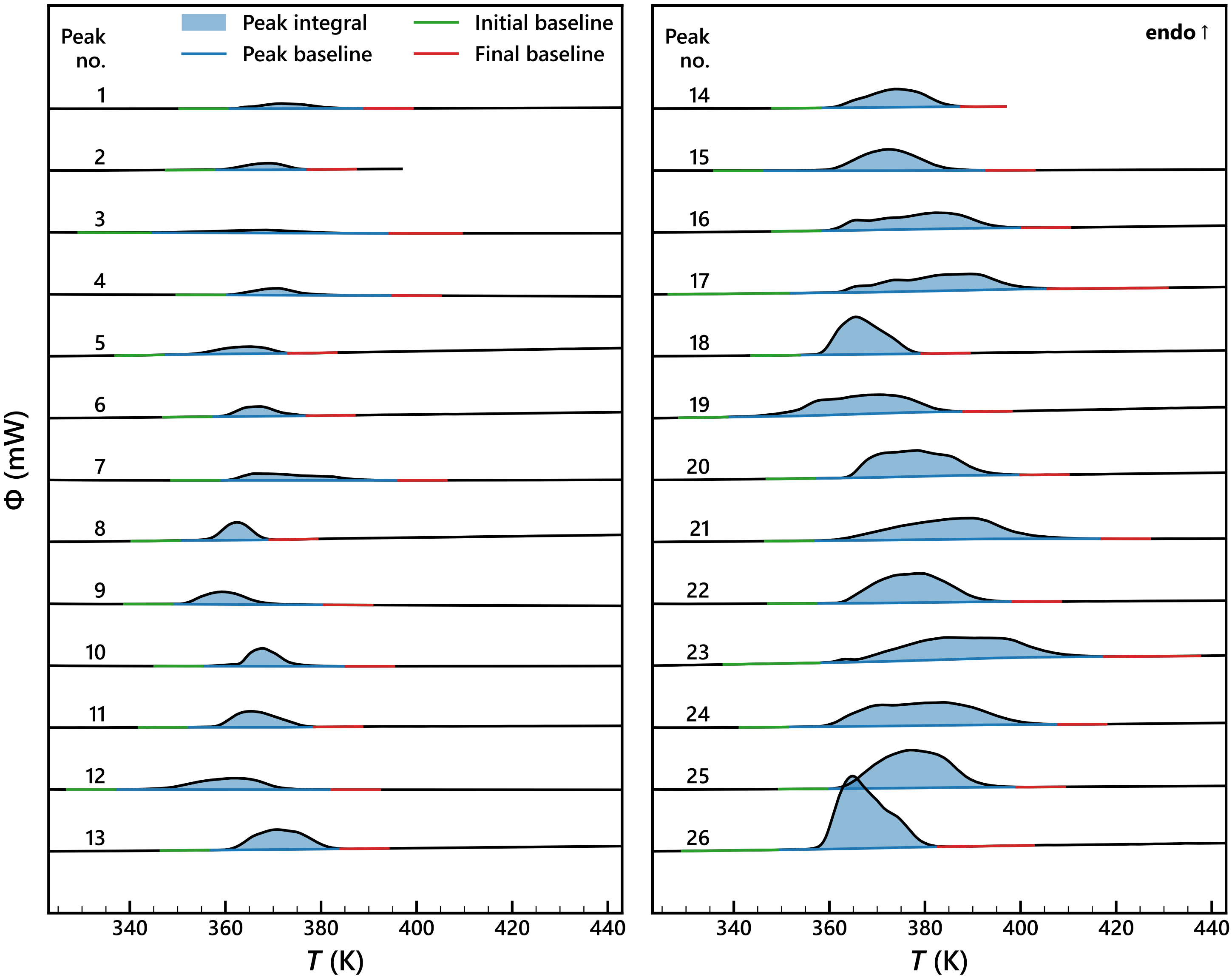

Supplementary Figure 19 FDSC thermograms (offset for clarity) of the $\alpha \rightarrow \beta$ solid–solid transition for the ChCl particles listed in table 1. All data was recorded at $1\,\mathrm{kK{\cdot}s^{-1}}$ and a sampling rate of 10 kHz. The integrated peak area (blue area), the peak baseline (blue line), the initial baseline, and final baseline (red line) are highlighted.

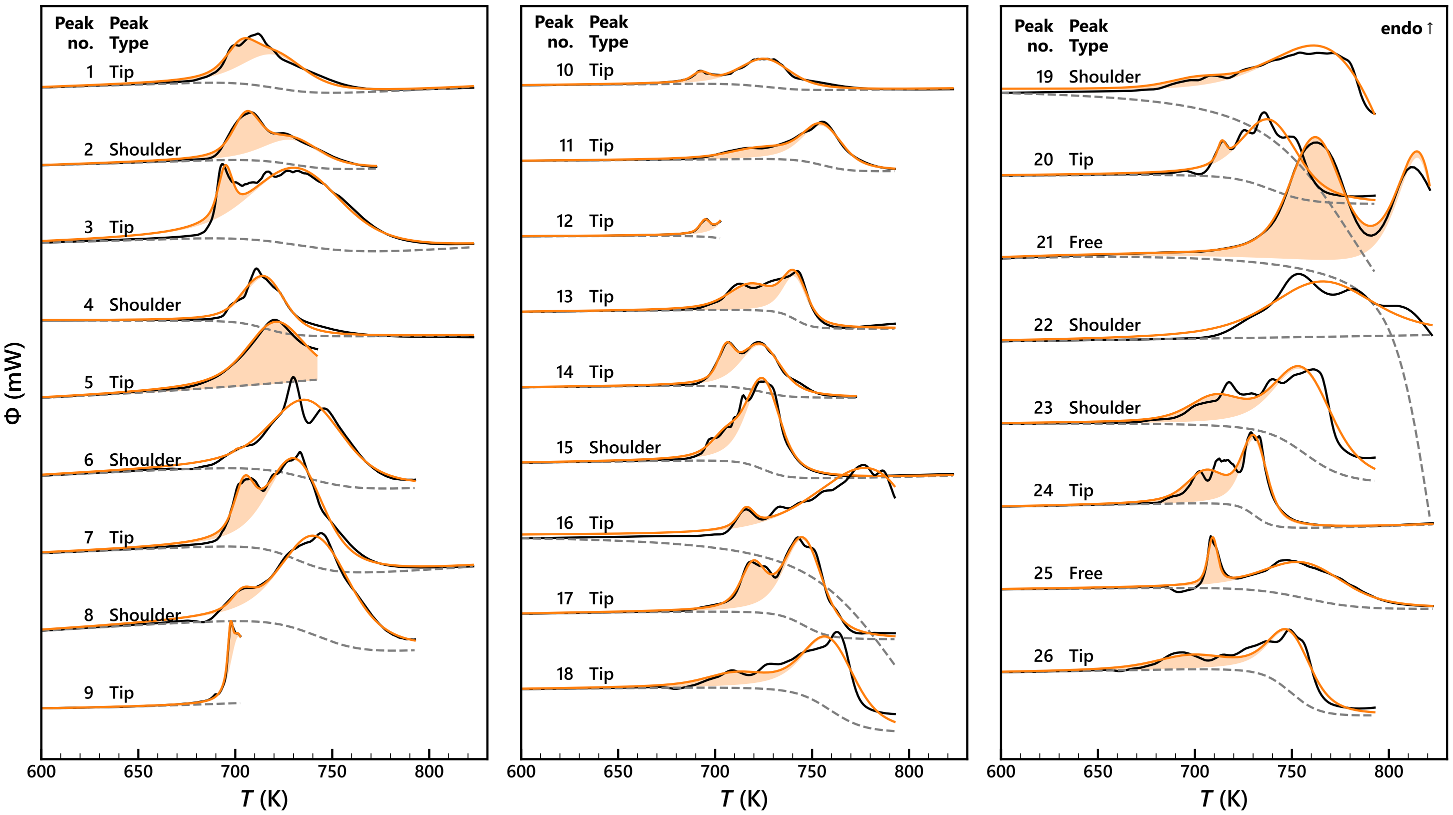

Supplementary Figure 20 FDSC thermograms (offset for clarity) of the high-temperature transitions for the ChCl particles listed in table 1. All data was recorded at a sampling rate of 10 kHz, the heating rate is listed in table 1 for each peak. Highlighted are the fit to the data using the melting + decomposition approach described in section Data acquisition and processing (orange line), the integrated melting peak area (orange area), the peak baseline (grey dashes), as well as the peak type qualification.

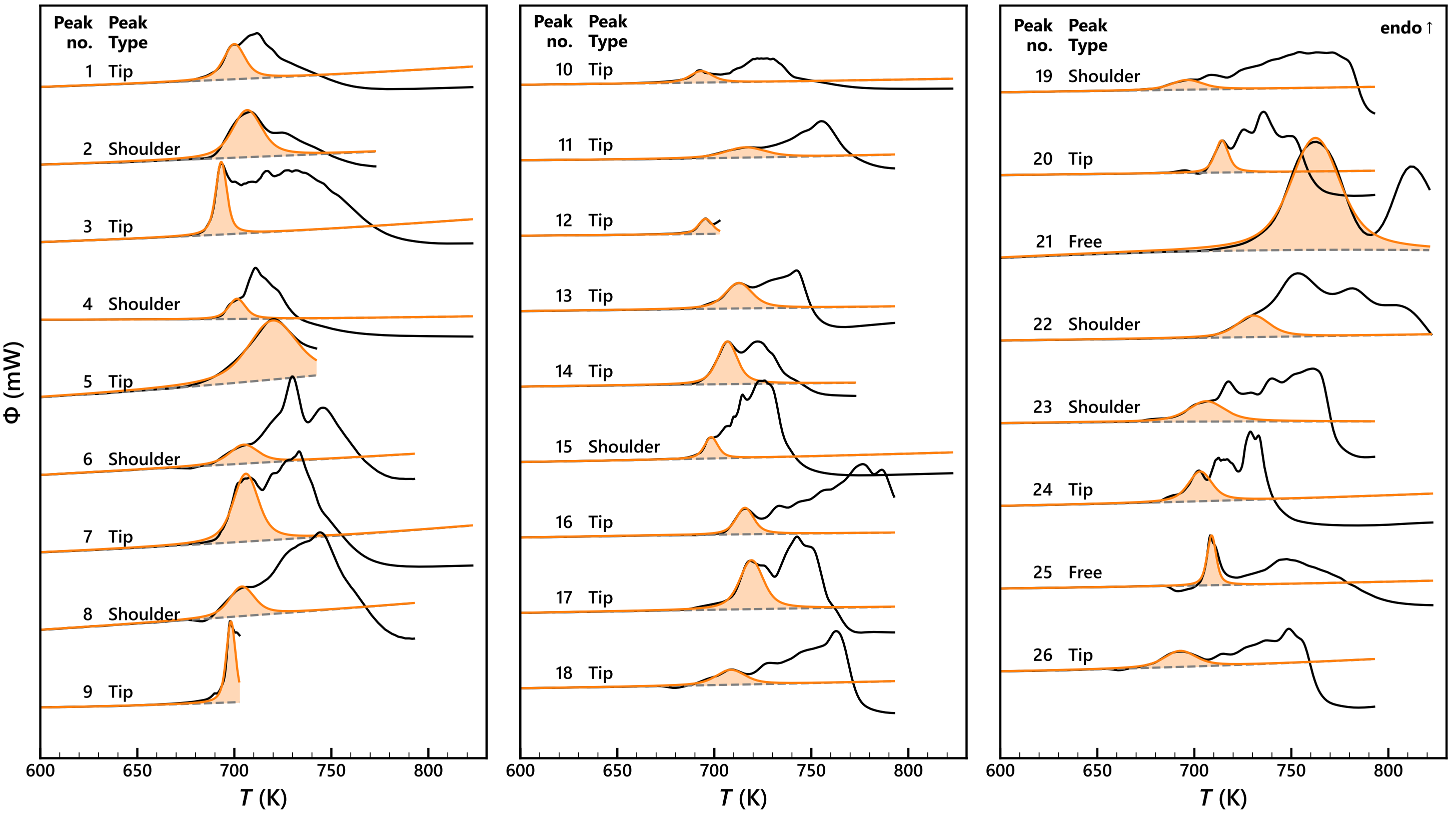

Supplementary Figure 21 FDSC thermograms (offset for clarity) of the high-temperature transitions for the ChCl particles listed in table 1. All data was recorded at a sampling rate of 10 kHz, the heating rate is listed in table 1 for each peak. Highlighted are the fit to the data using the single melting peak approach described in section Data acquisition and processing (orange line), the integrated melting peak area (orange area), the peak baseline (grey dashes), as well as the peak type qualification.

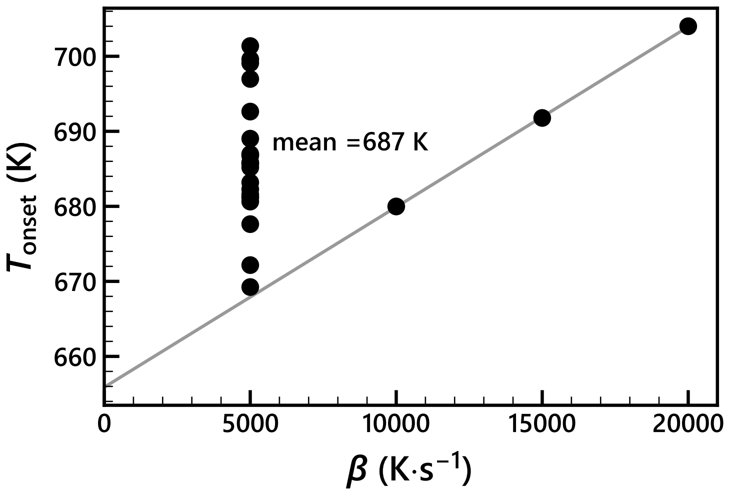

Supplementary Figure 22 Onset temperature (T_{onset}) as a function of heating rate (β) determined from the extrapolated peak onset. The mean displayed is for all samples measured at $\beta = 5000\,\mathrm{K{\cdot}s^{-1}}$, except for the outlier in 23. The line is a linear fit to all points with $\beta > 5000\,\mathrm{K{\cdot}s^{-1}}$. These trends are likely to change when more experiments are performed at $\beta > 5000\,\mathrm{K{\cdot}s^{-1}}$.

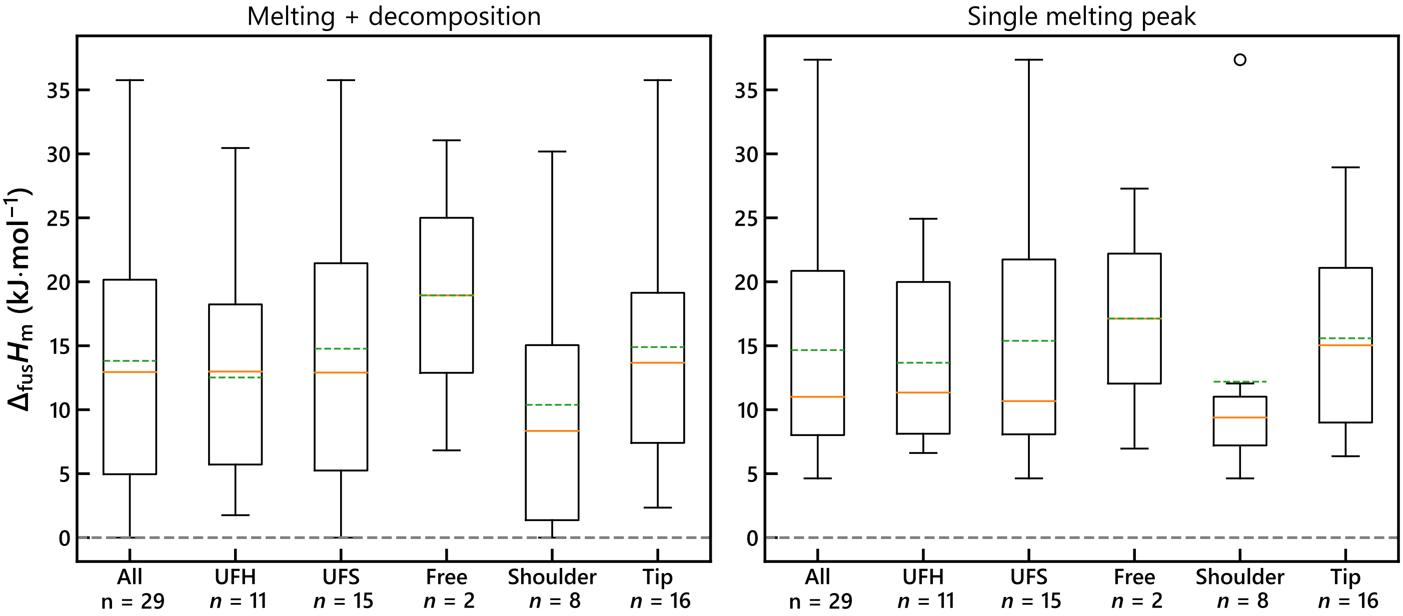

Supplementary Figure 23 Boxplots of $\Delta_{\mathrm{fus}}H_{\mathrm{m}}$ calculated from the melting + decomposition model (*left*) and single melting peak (*right*) for all samples, split by FDSC chip type (UFH or UFS, see section Equipment), and split by peak type as listed in Supplementary Table 1. Shown are the median (orange line), the mean (green dashes), the box ranging from the first to the third quartile, and the whiskers extending the box with 1.5 times the range of the box. Circles are outliers that fall beyond the whiskers. n is the number of peaks used to construct each boxplot.

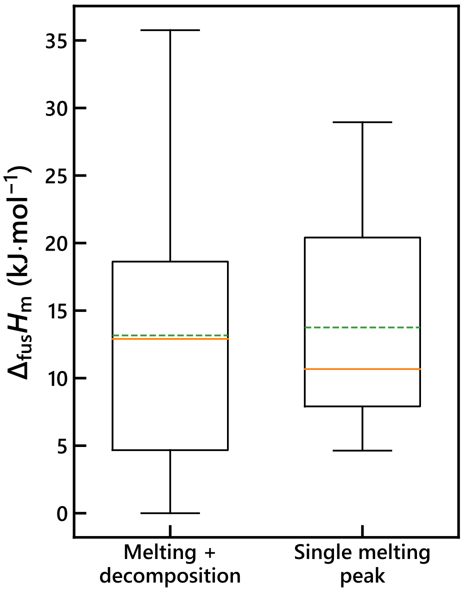

Supplementary Figure 24 Boxplots of $\Delta_{\mathrm{fus}}H_{\mathrm{m}}$ calculated from the melting + decomposition model and single melting peak, omitting the outlier (Supplementary Figure 23) for the shoulder peak type of the single melting peak model (peak 2 in Supplementary Table 1). Shown are the median (orange line), the mean (green dashes), the box ranging from the first to the third quartile, and the whiskers extending the box with 1.5 times the range of the box.

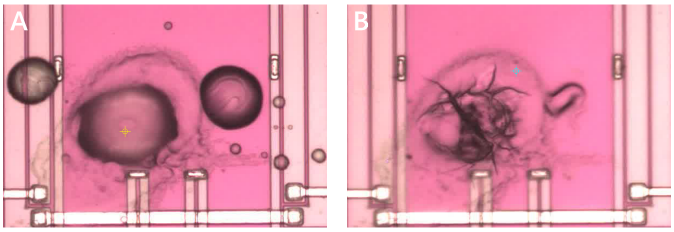

Supplementary Figure 25 Microscope images of choline chloride on silicon grease at 383 K (A) before heating, (B) after heating until 730 K at 100 K·s^{-1} and subsequent cooling while exposed to the µ-XRD beam.

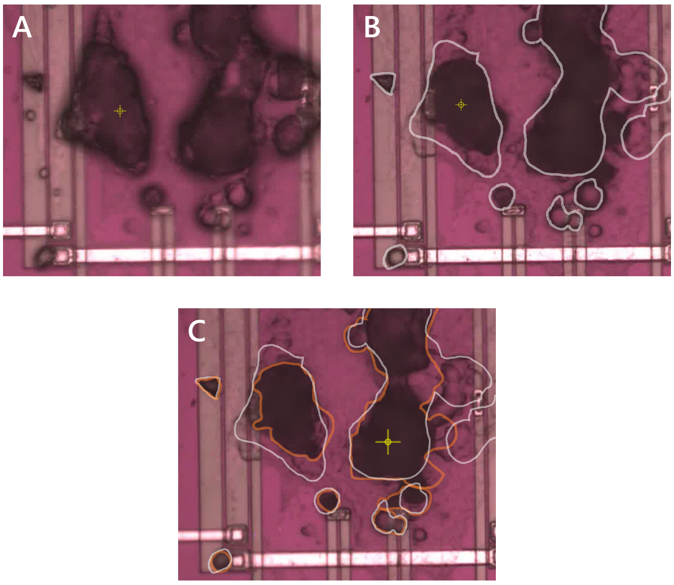

Supplementary Figure 26 Microscope images of choline chloride (ChCl) on silicon grease at 383 K (A) before heating, (B) after heating until 730 K at 2000 K·s^{-1} and subsequent cooling while exposed to the micro-XRD beam, and (C) after a second cycle. The contours of the ChCl particles in (A) and (B) are highlighted in white and orange, respectively.

Supplementary Tables

Supplementary Table 1 Fast differential scanning calorimeter results for several experiments on ChCl. The results are sorted by m, the mass of the ChCl particle as determined from the corresponding peak integral of the ChCl $\alpha \rightarrow \beta$ solid–solid transition ($\Delta_{\mathrm{trs}}H$) recorded at a heating rate (β) of $1\,\mathrm{kK{\cdot}s^{-1}}$ and $\Delta_{\mathrm{trs}}H_{\mathrm{m}} = 16.3\,\mathrm{kJ{\cdot}mol^{-1}}$ [3]. The ChCl molar fusion enthalpy ($\Delta_{\mathrm{fus}}H_{\mathrm{m}}$) was calculated from the peak integral of the first event, $\Delta_{\mathrm{trs}}H$, and $\Delta_{\mathrm{trs}}H_{\mathrm{m}}$ (section Data acquisition and processing). The peak type of the first event was qualified as Free, Tip, Shoulder, or Strange, in order to signify a freestanding peak, a peak with a clear maximum, a shoulder of a larger peak, or an unexpected peak shape, respectively. Finally, the commercial chip type is reported.

	m	$\Delta_{\mathrm{trs}}H$	β	T_{onset}	$\Delta_{\mathrm{fus}}H_{\mathrm{m}}$ [a]	$\Delta_{\mathrm{fus}}H_{\mathrm{m}}$ [b]	Peak type	Chip
Peak no.	ng	µJ	$\mathrm{kK{\cdot}s^{-1}}$	K	$\mathrm{kJ{\cdot}mol^{-1}}$	K	$\mathrm{kJ{\cdot}mol^{-1}}$	
1	2.6	0.31	5	683	20.7	21.0	Tip	UFH
2	2.7	0.32	5	683	30.2	37.4	Shoulder	UFS
3	2.8	0.33	5	680	17.8	21.3	Tip	UFH
4	3.0	0.35	5	685	1.8	8.3	Shoulder	UFH
5	3.7	0.43	10	679	35.8	28.9	Tip	UFS
6	3.8	0.45	5	681	0.2	10.4	Shoulder	UFS
7	5.0	0.59	5	686	13.7	24.9	Tip	UFH
8	5.2	0.60	5	681	4.7	10.7	Shoulder	UFS
9	5.6	0.66	5	686	2.3	9.7	Tip	UFH
10	5.9	0.69	5	677	7.9	13.3	Tip	UFH
11	7.5	0.87	5	685	21.7	16.8	Tip	UFS
12	8.1	0.94	5	681	4.6	7.9	Tip	UFH
13	11.0	1.28	5	692	30.5	19.0	Tip	UFH
14	11.1	1.30	5	689	13.7	23.1	Tip	UFS
15	13.3	1.55	5	685	13.0	6.6	Shoulder	UFH
16	15.5	1.81	5	699	5.8	9.1	Tip	UFS
17	17.2	2.01	5	701	18.2	20.4	Tip	UFS
18	17.6	2.06	5	686	12.9	7.6	Tip	UFS
19	18.7	2.18	5	672	12.0	7.4	Shoulder	UFS
20	22.1	2.58	5	699	3.0	8.6	Tip	UFS
21	22.4	2.62	20	704	31.1	27.3	Free	UFS
22	22.5	2.63	15	691	0.0	4.6	Shoulder	UFS
23	27.3	3.18	5	682	21.2	12.1	Shoulder	UFS
24	27.4	3.20	5	685	18.6	11.3	Tip	UFH
25	28.9	3.37	5	696	6.8	7.0	Free	UFH
26	35.7	4.17	5	669	11.2	6.4	Tip	UFS

[a] Fitted as melting + decomposition (section Data acquisition and processing)
[b] Fitted as single melting peak (section Data acquisition and processing)

Supplementary Table 2 Final values and uncertainties for $\Delta_{\mathrm{trs}}H_{\mathrm{m}}$, omitting the outlier (Supplementary Figure 23) for the shoulder peak type of the single melting peak model (peak 2 in Supplementary Table 1).

	T_{fus}[a] K	$\Delta_{\mathrm{fus}}H_{\mathrm{m}}$[a] kJ·mol^{-1}	T_{fus}[b] K	$\Delta_{\mathrm{fus}}H_{\mathrm{m}}$[b] kJ·mol^{-1}
median	686	12.9	685	10.7
mean	685	13.2	687	13.8
u_{sample}[c]	12	9.9	9	7.3
u_{mean}[d]	5	4.1	4	3.0

[a] Fitted as melting + decomposition (section Data acquisition and processing)
[b] Fitted as single melting peak (section Data acquisition and processing)
[c] Standard deviation
[d] Calculated from a two-sided confidence interval of 95%

Supplementary Table 3 The obtained μ-XRD results were animated to obtain an interactive overview of the Bragg-peaks with the temperature program. In addition to the temperature response of the FSC (top left), each recorded diffraction pattern is shown as raw detector counts (bottom left), as 1D-integration yielding the peak intensity I as a function of the modulus of the scattering vector q (top right), and all 1D-integrated data stacked over time with I as colour-map. Below we present an overview of which file corresponds to which experiment. The composition of the sample is expressed in mole fraction of ChCl (x_{ChCl}), the heating rate β of the experiment is also listed.

file	component 1	component 2	x_{ChCl}	β kK·s^{-1}
Supplementary Video 1		ChCl	1.0	1
Supplementary Video 2		ChCl	1.0	1
Supplementary Video 3[a]		ChCl	1.0	2
Supplementary Video 4		ChCl	1.0	5
Supplementary Video 5	Urea	ChCl	0.9	5
Supplementary Video 6	Urea	ChCl	0.8	5
Supplementary Video 7	Urea	ChCl	0.7	5
Supplementary Video 8	Ethylene glycol	ChCl	0.8	5

[a] Recorded on the same particle as Supplementary Video 2, after heating at 1 kK·s^{-1}.

Supplementary Table 4 The images obtained from the high-speed camera were combined with the temperature response of the FSC to obtain an interactive overview of the particle morphology with time and temperature. Below the files are listed together with the corresponding heating rate β of the experiment.

file	β kK·s^{-1}
Supplementary Video 9	0.1
Supplementary Video 10	1
Supplementary Video 11	2
Supplementary Video 12	5

Supplementary Table 5 Indirectly derived melting point (T_{fus}) and molar enthalpy of fusion ($\Delta_{fus}H_m$) of choline chloride (ChCl) in literature, estimated from experimental data on ChCl mixtures below the decomposition temperature[b] and assumptions regarding the thermodynamics of mixing. Ideal denotes ideal mixing entropy and $H^E = 0$, PC-SAFT is the acronym of the perturbed-chain statistical association fluid theory model that was used to calculate $\Delta_{mix}G$, in the last case H^E was assumed to be the same for all systems.

T_{fus} K	$\Delta_{fus}H_m$ kJ·mol^{-1}	Data source	Thermodynamics of mixing	Ref
597	4.30	SLE data	ideal	[14]
	7.77	water activity	PC-SAFT	[18]
	13.5[a]	$\Delta_{sol}H_m$	same for different solvents	[19]

[a] The original value (29.8 kJ·mol^{-1} [19]) was corrected for $\Delta_{trs}H_m$ of the $\alpha \rightarrow \beta$ solid–solid transition (16.3 kJ·mol^{-1} [3]) omitting $\Delta_S^L C_p$.
[b] $T_{dec} \approx 578$ K at zero heating rate [3].

Supplementary Table 6 Thermal properties of choline salts with different anions, for some of which plastic crystal behaviour was confirmed. Listed are the nature of the transition, the transision temperature (T_{trs}), the associated molar transition enthalpy ($\Delta_{trs}H_m$) and entropy ($\Delta_{trs}S_m$), and corresponding references. Additionally, we list references to solid-state NMR studies twoards the molecular motion within the crystal lattice before and after each transition.

Anion	Transition	T_{trs} K	$\Delta_{trs}H_m$ kJ·mol^{-1}	$\Delta_{trs}S_m$ J·mol^{-1}·K^{-1}	References Thermal properties	Solid-state NMR
Cl^-	fusion	597[a]	4.3[a]	7.2[a]	[14]	[20,21]
Cl^-	fusion	687	13.8	20.1	this work	[20,21]
Cl^-	II–I	352.2	16.3	46.3	[3]	[20,21]
Cl^-	II–I	351	16.5	47	[22]	[20,21]
Br^-	II–I	363	16.2	44.6	[22]	[20,21]
I^-	II–I	436	6.23	14.3	[22]	[20,21]
I^-	III–II	367	12.8	34.9	[22]	[20,21]
$[BF_4]^-$	fusion	485	3.1	6.4	[23]	[23]
$[BF_4]^-$	II–I	402	1.4	3.5	[23]	[23]
$[BF_4]^-$	III–II	268	9.3	34.7	[23]	[23]
$[H_2PO_4]^-$	fusion	392	1.4	3.5	[24]	
$[H_2PO_4]^-$	II–I	296			[24]	
$[ClO_4]^-$	fusion	560[b]	9[b]	16[b]	[25]	[25]
$[ClO_4]^-$	II–I	275	12.4	45.1	[25]	[25]

[a] derived indirectly from solid–liquid equilibrium data
[b] (partial) decomposition observed before melting, values are reported to be a rough estimate and the real enthalpy of fusion is expected to be significantly lower.

Supplementary Table 7 Supplementary data to construct Fig. 4 in the main text. Literature data of the liquidus temperatures (T_{liq}) for binary mixture urea + choline chloride (ChCl) at various mole fractions of ChCl (x_{ChCl}). Listed are the analysis technique, the calculated partial molar Gibbs energy ($G^{\text{E}}_{\text{m},i}$) in kJ·mol^{-1} of urea and ChCl based on the measured ChCl fusion properties in this work (FSC) and the estimated fusion properties from Fernandez *et al.* [14] (est), and the source papers (Ref). Analysis techniques are: freezing point determination (FP), hot-stage microscopy (HSM), differential scanning calorimetry (DSC), shaking-flask method (SF), melting point capillary (MPC), visual observation using an oil-bath (OB), and centrifuge method (CM)

x_{ChCl}	T_{liq}	Technique	$G^{\text{E}}_{\text{m,urea}}$	$G^{\text{E}}_{\text{m,ChCl}}$ (FSC)	$G^{\text{E}}_{\text{m,ChCl}}$ (est)	Ref
0.200	351.0	FP	-1.37			[26]
0.223	344.0	FP	-1.55			[26]
0.250	329.0	FP	-2.03			[26]
0.286	294.0	FP	-3.27			[26]
0.333	280.0	FP	-3.66			[26]
0.400	303.0	FP		-4.99	-0.31	[26]
0.500	329.0	FP		-4.12	0.30	[26]
0.556	357.0	FP		-3.03	1.10	[26]
0.625	417.0	FP		-2.60	0.92	[26]
0.100	398.2	HSM	0.05			[27]
0.200	376.9	HSM	-0.38			[27]
0.300	350.8	HSM	-0.99			[27]
0.330	305.9	HSM	-2.64			[27]
0.350	314.6	HSM		-3.99	0.57	[27]
0.400	341.8	HSM		-2.75	1.53	[27]
0.050	392.2	DSC	-0.35			[28]
0.100	378.2	DSC	-0.70			[28]
0.150	364.6	DSC	-1.03			[28]
0.200	337.7	DSC	-1.88			[28]
0.200	346.7	DSC	-1.53			[29]
0.250	311.2	DSC	-2.72			[29]
0.300	301.5	DSC	-2.93			[29]
0.350	300.6	DSC		-4.78	-0.08	[29]
0.400	316.8	DSC		-4.21	0.33	[29]
0.450	330.2	DSC		-3.76	0.64	[29]
0.500	338.2	DSC		-3.59	0.73	[29]
0.200	351.7	HSM	-1.34			[29]
0.250	336.2	HSM	-1.75			[29]
0.300	305.1	HSM	-2.78			[29]
0.350	304.3	HSM		-4.58	0.09	[29]
0.400	313.1	HSM		-4.42	0.16	[29]
0.450	334.3	HSM		-3.52	0.84	[29]
0.500	344.5	HSM		-3.23	1.03	[29]
0.147	371.9	DSC	-0.77			[30]
0.248	308.6	DSC	-2.83			[30]
0.298	303.0	DSC	-2.87			[30]

Continued on next page

Supplementary Table 7 – continued from previous page

x_{ChCl}	T_{liq}	Technique	$G^{\mathrm{E}}_{\mathrm{m,urea}}$	$G^{\mathrm{E}}_{\mathrm{m,ChCl}}$ (FSC)	$G^{\mathrm{E}}_{\mathrm{m,ChCl}}$ (est)	Ref
0.332	291.8	DSC	-3.19			[30]
0.398	316.0	DSC		-4.24	0.30	[30]
0.448	330.9	DSC		-3.71	0.69	[30]
0.498	342.1	DSC		-3.36	0.93	[30]
0.549	349.5	DSC		-3.21	1.00	[30]
0.101	381.8	MPC	-0.56			[17]
0.200	355.5	MPC	-1.20			[17]
0.596	380.4	MPC		-2.94	0.95	[17]
0.698	438.7	MPC		-2.68	0.62	[17]
0.796	475.6	MPC		-2.64	0.28	[17]
0.890	539.6	MPC		-2.11	0.16	[17]
0.307	321.3	OB	-2.12			[17]
0.391	294.6	OB		-5.39	-0.62	[17]
0.493	331.8	OB		-3.92	0.47	[17]
0.260	325.0	CM	-2.15			[31]
0.273	319.2	CM	-2.33			[31]
0.306	314.6	CM	-2.39			[31]
0.313	311.2	CM	-2.50			[31]
0.317	316.4	CM	-2.28			[31]
0.324	309.4	CM	-2.53			[31]
0.329	306.7	CM	-2.61			[31]
0.340	302.7	CM	-2.73			[31]
0.342	301.4	CM	-2.78			[31]
0.348	301.6	CM		-4.71	-0.02	[31]
0.357	306.5	CM		-4.50	0.14	[31]
0.374	310.1	CM		-4.42	0.19	[31]
0.378	313.5	CM		-4.25	0.32	[31]
0.385	311.0	CM		-4.44	0.16	[31]
0.392	316.6	CM		-4.17	0.37	[31]
0.421	319.5	CM		-4.19	0.32	[31]
0.430	324.6	CM		-3.96	0.50	[31]

Supplementary References

[1] S. van Herwaarden, E. Iervolino, F. van Herwaarden, T. Wijffels, A. Leenaers, V. Mathot, *Thermochim. Acta* **2011**, *522*, 46–52.

[2] J. E. K. Schawe, S. Pogatscher in *Material Characterization by Fast Scanning Calorimetry: Practice and Applications*, Springer International Publishing, Cham, **2016**, pp. 3–80.

[3] A. I. M. C. Lobo Ferreira, S. M. Vilas-Boas, R. M. A. Silva, M. A. R. Martins, D. O. Abranches, P. C. R. Soares-Santos, F. A. Almeida Paz, O. Ferreira, S. P. Pinho, L. M. N. B. F. Santos, J. A. P. Coutinho, *Phys. Chem. Chem. Phys.* **2022**, *24*, 14886–14897.

[4] M. Newville, R. Otten, A. Nelson, T. Stensitzki, A. Ingargiola, D. Allan, A. Fox, F. Carter, Michał, R. Osborn, D. Pustakhod, lneuhaus, S. Weigand, A. Aristov, Glenn, C. Deil, Mark, A. L. R. Hansen, G. Pasquevich, L. Foks, N. Zobrist, O. Frost, Stuermer, azelcer, A. Polloreno, A. Persaud, J. H. Nielsen, M. Pompili, S. Caldwell, A. Hahn, *lmfit/lmfit-py: 1.1.0*, **2022**, `https://doi.org/10.5281/zenodo.598352`.

[5] M. Rosenthal, D. Doblas, J. J. Hernandez, Y. I. Odarchenko, M. Burghammer, E. Di Cola, D. Spitzer, A. E. Antipov, L. S. Aldoshin, D. A. Ivanov, *J. Synchrotron Radiat.* **2014**, *21*, 223–228.

[6] M. Rosenthal, A. P. Melnikov, A. A. Rychkov, D. Doblas, D. V. Anokhin, M. Burghammer, D. A. Ivanov in *Design of an In Situ Setup Combining Nanocalorimetry and Nano- or Micro-focus X-Ray Scattering to Address Fast Structure Formation Processes*, Springer International Publishing, Cham, **2016**, pp. 299–326.

[7] M. Rosenthal, A. P. Melnikov, M. Burghammer, D. A. Ivanov, *European Polymer Journal* **2017**, *94*, 517–523.

[8] M. Vlasova, *Theses*, Université de Haute Alsace - Mulhouse, **2019**.

[9] A. P. Melnikov, M. Rosenthal, D. A. Ivanov, *ACS Macro Letters* **2018**, *7*, 1426–1431.

[10] R. L. Collin, *J. Am. Chem. Soc.* **1957**, *79*, 6086–6086.

[11] G. Ashiotis, A. Deschildre, Z. Nawaz, J. P. Wright, D. Karkoulis, F. E. Picca, J. Kieffer, *Journal of Applied Crystallography* **2015**, *48*, 510–519.

[12] J. Kieffer, D. Karkoulis, *J. Phys. Conf. Ser.* **2013**, *425*, 202012.

[13] J. Kieffer, V. Valls, N. Blanc, C. Hennig, *J. Synchrotron Radiat.* **2020**, *27*, 558–566.

[14] L. Fernandez, L. P. Silva, M. A. R. Martins, O. Ferreira, J. Ortega, S. P. Pinho, J. A. Coutinho, *Fluid Phase Equilib.* **2017**, *448*, 9–14.

[15] A. Abdelaziz, D. H. Zaitsau, T. A. Mukhametzyanov, B. N. Solomonov, P. Cebe, S. P. Verevkin, C. Schick, *Thermochim. Acta* **2017**, *657*, 47–55.

[16] A. van den Bruinhorst, M. Costa Gomes, *Curr. Opin. Green Sustain. Chem.* **2022**, 100659.

[17] L. P. Silva, C. F. Araújo, D. O. Abranches, M. Melle-Franco, M. A. R. Martins, M. M. Nolasco, P. J. A. Ribeiro-Claro, S. P. Pinho, J. A. Coutinho, *Phys. Chem. Chem. Phys.* **2019**, *21*, 18278–18289.

[18] S. M. Vilas-Boas, D. O. Abranches, E. A. Crespo, O. Ferreira, J. A. Coutinho, S. P. Pinho, *J. Mol. Liq.* **2020**, *300*, 112281.

[19] P. López-Porfiri, J. F. Brennecke, M. Gonzalez-Miquel, *J. Chem. Eng. Data* **2016**, *61*, 4245–4251.

[20] D. Wemmer, V. Petrouleas, N. Panagiotopoulos, S. E. Filippakis, R. M. Lemmon, *J. Phys. Chem.* **1983**, *87*, 999–1003.

[21] T. Pratum, M. Klein, *J. Magn. Reson.* **1989**, *81*, 350–370.

[22] V. Petrouleas, R. M. Lemmon, *J. Chem. Phys.* **1978**, *69*, 1315.

[23] H. Ishida, T. Asaji, Y. Furukawa, R. Ikeda, *Chem. Lett.* **1993**, *22*, 1207–1210.

[24] M. Yoshizawa-Fujita, K. Fujita, M. Forsyth, D. R. MacFarlane, *Electrochem. commun.* **2007**, *9*, 1202–1205.

[25] H. Ishida, M. Kato, H. Ono, R. Ikeda, *Zeitschrift für Naturforsch. A* **1997**, *52*, 637–639.

[26] A. P. Abbott, G. Capper, D. L. Davies, R. K. Rasheed, V. Tambyrajah, *Chem. Commun.* **2003**, 70–71.

[27] M. Gilmore, M. Swadzba-Kwasny, J. D. Holbrey, *J. Chem. Eng. Data* **2019**, *64*, 5248–5255.

[28] K.-S. Kim, B. H. Park, *J. Chem. Eng. Japan* **2015**, *48*, 881–884.

[29] X. Meng, K. Ballerat-Busserolles, P. Husson, J.-M. Andanson, *New J. Chem.* **2016**, *40*, 4492–4499.

[30] H. G. Morrison, C. C. Sun, S. Neervannan, *Int. J. Pharm.* **2009**, *378*, 136–9.

[31] A. van den Bruinhorst, L. J. B. M. Kollau, M. C. Kroon, J. Meuldijk, R. Tuinier, A. C. C. Esteves, *J. Chem. Phys.* **2018**, *149*, 224505.
