# Supplementary material for: Defying decomposition: the curious case of choline chloride
Source: Nat Commun. 2023 Oct 21;14:6684. doi: 10.1038/s41467-023-42267-6 (PMC10590384; doi:10.1038/s41467-023-42267-6)
Supplement: Supplementary file 3 — Description of Additional Supplementary Files [file 41467_2023_42267_MOESM3_ESM.pdf]

Description of Additional Supplementary Files

Supplementary Movie 1
Description: Animated μ-XRD results for pure choline chloride heated at 1000 K s^{-1} showing the recorded Bragg-peaks for the whole temperature program. **(top left)** temperature (*T*) as a function of time (*t*), **(top right)** 1D integration of the diffraction pattern at each *t* and *T* yielding peak intensity I as a function of the modulus of the scattering vector *q*, **(bottom left)** raw detector counts at each *t* and *T*, and **(top right)**, all 1D-integrated data stacked over time with I as colour-map.

Supplementary Movie 2
Description: Animated μ-XRD results for pure choline chloride (different particle than in Supplementary Movie 1) heated at 1000 K s^{-1} showing the recorded Bragg-peaks for the whole temperature program. **(top left)** temperature (*T*) as a function of time (*t*), **(top right)** 1D integration of the diffraction pattern at each *t* and *T* yielding peak intensity I as a function of the modulus of the scattering vector *q*, **(bottom left)** raw detector counts at each *t* and *T*, and **(top right)**, all 1D-integrated data stacked over time with I as colour-map.

Supplementary Movie 3
Description: Animated μ-XRD results for pure choline chloride (same particle as shown in Supplementary Movie 2, but after one heating–cooling cycle) heated at 2000 K s^{-1} showing the recorded Bragg-peaks for the whole temperature program. **(top left)** temperature (*T*) as a function of time (*t*), **(top right)** 1D integration of the diffraction pattern at each *t* and *T* yielding peak intensity I as a function of the modulus of the scattering vector *q*, **(bottom left)** raw detector counts at each *t* and *T*, and **(top right)**, all 1D-integrated data stacked over time with I as colour-map.

Supplementary Movie 4
Description: Animated μ-XRD results for pure choline chloride (different particle than in Supplementary Movie 1–3) heated at 5000 K s^{-1} showing the recorded Bragg-peaks for the whole temperature program. **(top left)** temperature (*T*) as a function of time (*t*), **(top right)** 1D integration of the diffraction pattern at each *t* and *T* yielding peak intensity I as a function of the modulus of the scattering vector *q*, **(bottom left)** raw detector counts at each *t* and *T*, and **(top right)**, all 1D-integrated data stacked over time with I as colour-map.

Supplementary Movie 5
Description: Animated μ-XRD results for a mixture of urea + choline chloride x_{ChCl}=0.9 heated at 5000 K s^{-1} showing the recorded Bragg-peaks for the whole temperature program. **(top left)** temperature (*T*) as a function of time (*t*), **(top right)** 1D integration of the diffraction pattern at each *t* and *T* yielding peak intensity I as a function of the modulus of the scattering vector *q*, **(bottom left)** raw detector counts at each *t* and *T*, and **(top right)**, all 1D-integrated data stacked over time with I as colour-map.

Supplementary Movie 6
Description: Animated μ-XRD results for a mixture of urea + choline chloride x_{ChCl}=0.8 heated at 5000 K s^{-1} showing the recorded Bragg-peaks for the whole temperature program. **(top left)** temperature (*T*) as a function of time (*t*), **(top right)** 1D integration of the diffraction

pattern at each *t* and *T* yielding peak intensity I as a function of the modulus of the scattering vector *q*, **(bottom left)** raw detector counts at each *t* and *T*, and **(top right)**, all 1D-integrated data stacked over time with I as colour-map.

Supplementary Movie 7 Animated μ-XRD results for a mixture of urea + choline chloride x_{ChCl}=0.7 heated at 5000 K s^{-1} showing the recorded Bragg-peaks for the whole temperature program. **(top left)** temperature (*T*) as a function of time (*t*), **(top right)** 1D integration of the diffraction pattern at each *t* and *T* yielding peak intensity I as a function of the modulus of the scattering vector *q*, **(bottom left)** raw detector counts at each *t* and *T*, and **(top right)**, all 1D-integrated data stacked over time with I as colour-map.

Supplementary Movie 8 Animated μ-XRD results for a mixture of ethylene glycol + choline chloride x_{ChCl}=0.8 heated at 5000 K s^{-1} showing the recorded Bragg-peaks for the whole temperature program. **(top left)** temperature (*T*) as a function of time (*t*), **(top right)** 1D integration of the diffraction pattern at each *t* and *T* yielding peak intensity I as a function of the modulus of the scattering vector *q*, **(bottom left)** raw detector counts at each *t* and *T*, and **(top right)**, all 1D-integrated data stacked over time with I as colour-map.

Supplementary Movie 9 Particle morphology of pure choline chloride heated at 100 K s^{-1} as a function of temperature (*T*) and time (*t*) showing **(left)** high-speed camera images and **(right)** the FSC response.

Supplementary Movie 10 Particle morphology of pure choline chloride heated at 1000 K s^{-1} as a function of temperature (*T*) and time (*t*) showing **(left)** high-speed camera images and **(right)** the FSC response.

Supplementary Movie 11 Particle morphology of pure choline chloride heated at 2000 K s^{-1} as a function of temperature (*T*) and time (*t*) showing **(left)** high-speed camera images and **(right)** the FSC response.

Supplementary Movie 12 Particle morphology of pure choline chloride heated at 5000 K s^{-1} as a function of temperature (*T*) and time (*t*) showing **(left)** high-speed camera images and **(right)** the FSC response.
